# Exploratory analysis of gene aberrations and chemotherapy response: findings from a real-world database in Japan

**DOI:** 10.1038/s41416-025-03281-1

**Published:** 2025-12-22

**Authors:** Naoya Ishibashi, Takashi Kamatani, Satoru Aoyama, Masanori Tokunaga, Yusuke Kinugasa, Sadakatsu Ikeda

**Affiliations:** 1 Department of Precision Cancer Medicine, Institute of Science Tokyo Hospital, Tokyo, Japan; 2 Department of Gastrointestinal Surgery, Institute of Science Tokyo, Tokyo, Japan; 3 Department of AI Technology Development, M&D Data Science Center, Institute of Integrated Research, Institute of Science Tokyo, Tokyo, Japan

**Keywords:** Targeted therapies, Chemotherapy

## Abstract

**Background:**

Chemotherapy selection traditionally relies on tumor tissue of origin. However, since genetic alterations drive tumor behavior, it remains unclear if mutations can better predict response. We hypothesized that genetic aberrations might influence chemotherapy outcomes more than tissue origin.

**Methods:**

We retrospectively analyzed 15,474 Japanese patients with solid tumors who underwent comprehensive genomic profiling (CGP) and received cytotoxic chemotherapy. Genetic alterations and tumor origin were evaluated for objective response rate (ORR) and time to next treatment (TNT). Gene mutations were assessed across five chemotherapy classes: platinum-based, alkylating agents, antimetabolites, microtubule inhibitors, and topoisomerase inhibitors.

**Results:**

Genomic alteration data alone did not surpass organ-based models in predicting response. For platinum-based agents, the gene-only model had an AUC of 0.575 versus 0.604 for the organ-only model. A combined gene-organ model yielded an AUC of 0.618 (*P* < 0.01). Certain gene-organ interactions were associated with improved outcomes. For example, APC-mutated colorectal cancer showed higher ORR and prolonged TNT (hazard ratio, 0.82; 95% CI, 0.73–0.92; *P* < 0.001) for platinum-based drugs.

**Conclusions:**

While genetic alterations alone did not outperform tumor origin as a predictor, incorporating both may improve exploratory predictions of chemotherapy response. These exploratory findings require prospective validation before any clinical application.

## Introduction

Surgical resection, radiotherapy, and chemotherapy have long been the mainstays of cancer treatment, with chemotherapy serving as a key option for patients with unresectable or metastatic solid tumors. Conventionally, chemotherapy regimens are chosen based on the primary tumor site or histopathological type, guided by evidence from previous clinical trials. Most cytotoxic anticancer agents exert antitumor effects by inhibiting nucleic acid synthesis and disrupting DNA replication and transcription, ultimately impairing cell division and inducing apoptosis [1–3]. Nevertheless, while some patients respond favorably to chemotherapy, others derive little benefit or experience severe adverse effects, even when receiving the same drug for the same cancer type.

Recent advances in cancer genomics have shown that cancer is fundamentally a genetic disease. Next-generation sequencing (NGS) and pan-cancer genome projects, such as The Cancer Genome Atlas (TCGA), have identified numerous driver genes and functional pathways, leading to the development of molecularly targeted therapies and novel molecular classifications of cancer [4–8]. Precision oncology, which tailors treatments based on tumor genomic profiles, has demonstrated clinical utility across various cancer subtypes in multiple trials and is now integrated into routine practice [9–11]. However, chemotherapy selection largely continues to rely on traditional factors—namely, the organ of origin and histopathological characteristics—with cytotoxic agents remaining key drugs. Whether genetic alterations can more accurately predict chemotherapy response than these conventional factors remain unclear.

In this study, we hypothesized that chemotherapy response might be associated more by genetic aberrations than by the tumor’s tissue of origin or pathology. To test this hypothesis, we analyzed a nationwide real-world database established by the Japanese national central data center, the Center for Cancer Genomics and Advanced Therapy (C-CAT). Our aim was to determine whether such a hypothesis would be supported, by examining associations between specific gene mutations and cytotoxic chemotherapy outcomes across multiple organs.

## Materials and methods

### Data collection

This retrospective, real-world study analyzed clinical and genetic variant data from diverse cancer types registered in the C-CAT database. Several comprehensive genomic profiling (CGP) assays are approved for use across all solid tumors in Japan. These include two tissue-based CGP tests—the OncoGuide™ NCC Oncopanel System (tumor/normal paired, Sysmex Co., Ltd., Kobe, Japan) and FoundationOne® CDx (tumor-only)—as well as one liquid biopsy-based test, FoundationOne® Liquid CDx (Foundation Medicine Inc., Cambridge, USA). Under Japan’s national health insurance system, CGP testing is limited to patients with unresectable or metastatic solid tumors who have completed or are approaching the completion of standard therapy and are considered potential candidates for subsequent chemotherapy based on CGP results [12–14]. Accordingly, circulating tumor DNA was typically collected at the time of disease progression following standard treatment. For tissue-based assays, archived tumor samples were generally used, or alternatively, a new biopsy may be performed and submitted for CGP testing. This study was approved by the Institutional Review Board of our institution (No. M2021-379) and submitted to the C-CAT Information Utilization Review Board (Control Number: CDU2022-034N), which authorized the secondary use of data. Written informed consent for data registration in C-CAT and for the secondary use of clinical and genomic data was obtained from all patients at the time of enrollment. This study was performed in accordance with the Declaration of Helsinki and the Strengthening the Reporting of Observational Studies in Epidemiology (STROBE) guidelines.

### Data analysis

Due to the retrospective nature of the study, patients were not randomized. Instead, group allocation was based on pre-existing treatment regimens as recorded in the C-CAT database. We defined the objective response rate (ORR) as the proportion of patients achieving a partial or complete response to chemotherapy, as assessed by the treating physician. Chemotherapeutic agents were categorized into five classes based on their mechanisms of action: platinum-based drugs, alkylating agents, antimetabolites, microtubule inhibitors, and topoisomerase inhibitors. Real-world time to next treatment (TNT) was defined as the interval from the initiation of one treatment regimen until the start of the subsequent treatment. To focus on clinically relevant variants, we included only those classified in the C-CAT database as “oncogenic,” “pathogenic,” “likely oncogenic,” or “likely pathogenic” and excluded variants of unknown significance. Genetic alterations (e.g., missense or nonsense mutations, insertions/deletions, amplifications, losses, and rearrangements) were collectively labeled as “mutation positive”.

### Statistical analysis

Differences in continuous variables between two groups were assessed using the Mann-Whitney U test, and differences in categorical variables were evaluated using the chi-squared test for independence. We performed multivariable analyses to explore associations between clinical and genetic variables and treatment response. Logistic regression analysis was performed to evaluate the association between clinical/genetic variables and ORR, with results reported as odds ratios (ORs) and 95% confidence intervals (CIs). We performed Kaplan-Meier analyses to compare survival times between groups with and without specific gene alterations, and estimated hazard ratios (HRs) for time-to-event outcomes using Cox proportional hazards models. DeLong’s test was used to compare the areas under the receiver operating characteristic (ROC) curves. Statistical significance was set at *p* < 0.05. All analyses were performed using R software version 4.3.0 (R Foundation for Statistical Computing, Vienna, Austria).

## Results

### Overview of real-world data

Between June 2019 and June 2022, 35,377 patients with advanced solid cancers were registered in the C-CAT database. Of these, 15,474 patients, who underwent a total of 46,471 chemotherapy regimens, met the criteria for our analysis. (Supplementary Fig. 1 shows flow diagram of this analysis; cases with incomplete data were excluded from the analysis, and the reasons for exclusion were documented.) Table 1 summarizes the main patient characteristics. Subject demographics, including age and sex were recorded. The cohort comprised 7884 males and 7590 females with a mean age of 61 years. Common cancer types were colorectal(bowel), pancreatic, breast, esophageal/gastric, prostate, lung, ovarian, biliary tract, and head and neck cancers. The most frequently used CGP test was FoundationOne® CDx (73.5%), followed by FoundationOne® Liquid CDx and the NCC Oncopanel. Ninety-six percent of patients had an Eastern Cooperative Oncology Group (ECOG) Performance Status (PS) of 0 or 1, consistent with CGP eligibility criteria in Japan. Chemotherapeutic agents were classified into the following five categories: platinum-based drugs (e.g., cisplatin, carboplatin, oxaliplatin), alkylating agents (e.g., cyclophosphamide, ifosfamide), antimetabolites (e.g., fluorouracil, gemcitabine), microtubule inhibitors (e.g., paclitaxel, docetaxel, eribulin), and topoisomerase inhibitors (e.g., irinotecan, doxorubicin, etoposide). (Supplementary Table 1 provides 5 categories of chemotherapy drug class and cancers included in analysis) The CGP assays collectively examined 328 genes. (Supplementary Table 2 provides full list of genes included in comprehensive genomic profiling testing).

**Table.1:** Patients Characteristics Included in Analysis

	Overall
*n*	15,474
Age (mean (SD))	61.90 (11.82)
Sex = Female (%)	7590 (49.1)
ECOG-PS (%)	
0	8846 (57.2)
1	5997 (38.8)
2	427 (2.8)
3	54 (0.3)
4	5 (0.0)
Unknown	145 (0.9)
No. of CGP (%)	
NCC Oncopanel	1984 (12.8)
FoundationOne CDx	11,378 (73.5)
FoundationOneLiquid CDx	2112 (13.6)
No. of tumor samples(%)	
Tissue	
Primary Site	﻿8694 (56.2)
Metastatic Site	4645 (30.0)
Unknown	23 (0.1)
ctDNA	2112 (13.6)
No. of Organ (%)	
ADRENAL_GLAND	14 (0.1)
AMPULLA_OF_VATER	93 (0.6)
BILIARY_TRACT	872 (5.6)
BLADDER	251 (1.6)
BONE	44 (0.3)
BOWEL	3276 (21.2)
BRAIN	50 (0.3)
BREAST	1456 (9.4)
CERVIX	338 (2.2)
EYE	2 (0.0)
HEAD_NECK	478 (3.1)
KIDNEY	125 (0.8)
LIVER	144 (0.9)
LUNG	1254 (8.1)
MM	44 (0.3)
OVARY	914 (5.9)
PANCREAS	2202 (14.2)
PENIS	6 (0.0)
PERITONEUM	116 (0.7)
PLEURA	37 (0.2)
PNS	13 (0.1)
PROSTATE	1256 (8.1)
SKIN	78 (0.5)
SOFT_TISSUE	465 (3.0)
STOMACH	1332 (8.6)
TESTIS	20 (0.1)
THYMUS	100 (0.6)
THYROID	61 (0.4)
UTERUS	419 (2.7)
VULVA	14 (0.1)

### Association between gene mutations and chemotherapy response

We performed univariate and multivariable analyses to evaluate the relationship between gene mutations and chemotherapy response across different cancer types. Only gene alterations with a prevalence >10% in the relevant dataset for each drug class were included.

**Fig. 1:**
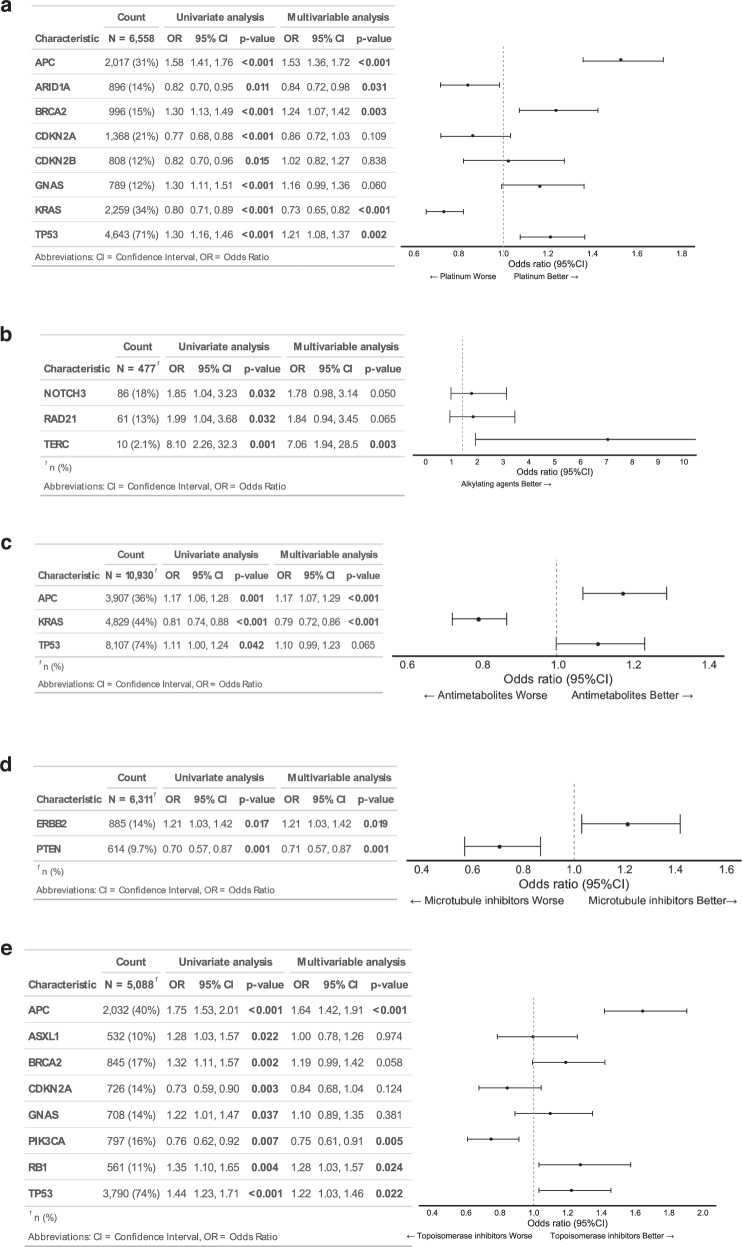
Multivariable analysis of overall response rate association between chemotherapy response and gene alterations. The analysis was conducted for five drug categories: **a** Platinum-based drugs, **b** Alkylating agents, **c** Antimetabolites, **d** Microtubule inhibitors, **e** Topoisomerase inhibitors.

In univariate analysis of platinum-based drugs, *APC* alterations were associated with an ORR of 39.8% in the mutated group, compared with 32.5% in the wild-type group (OR: 1.58, 95% CI: 1.46–1.76; *p* < 0.001). Other alterations correlated with higher ORRs with platinum agents included *BRCA2* (37.8% vs. 31.8%; OR: 1.30, 95% CI: 1.13–1.49; *p* < 0.001), *GNAS* (37.9% vs. 32.0%; OR: 1.30, 95% CI: 1.11–1.51; *p* < 0.001), and *TP53* (34.4% vs. 28.7%; OR: 1.30, 95% CI: 1.16–1.46; *p* < 0.001). Conversely, *ARID1A* (29.0% vs. 33.3%; OR: 0.82, 95% CI: 0.70–0.95; *p* = 0.011), *CDKN2A* (28.4% vs. 33.9%; OR: 0.77, 95% CI: 0.68–0.88; *p* < 0.001), *CDKN2B* (29.0% vs. 33.2%; OR: 0.82, 95% CI: 0.70–0.96; *p* = 0.015), and *KRAS* (29.5% vs. 34.4%; OR: 0.80, 95% CI: 0.71–0.89; *p* < 0.001) were associated with lower ORRs. In multivariable logistic regression, *APC* (OR: 1.53, 95% CI: 1.36–1.72; *p* < 0.001), *BRCA2* (OR: 1.24, 95% CI: 1.07–1.42; *p* = 0.003), and *TP53* (OR: 1.21, 95% CI: 1.08–1.37; *p* = 0.002) remained significantly associated with higher ORRs to platinum-based drugs. In contrast, *ARID1A* (OR: 0.84, 95% CI: 0.72–0.98; *p* = 0.031) and *KRAS* (OR: 0.73, 95% CI: 0.65–0.82; *p* < 0.001) were associated with lower ORRs (Fig. 1a).

Similar analyses were performed for other drug classes. For alkylating agents, *TERC* mutations significantly increased the ORR (OR: 7.06, 95% CI: 1.94–28.5; *p* = 0.003). (Fig. 1b) Results for antimetabolites were analogous to those for platinum-based drugs; *APC* mutations were linked to a higher ORR (OR: 1.17, 95% CI: 1.06–1.29; *p* < 0.001), whereas *KRAS* mutations lowered the ORR (OR: 0.79, 95% CI: 0.72–0.86; p < 0.001). (Fig. 1c) For microtubule inhibitors, *ERBB2* alterations correlated with a higher ORR (OR: 1.21, 95% CI: 1.03–1.42; *p* = 0.019), while *PTEN* mutations were associated with a lower ORR (OR: 0.71, 95% CI: 0.57–0.87; *p* = 0.001). (Fig. 1d) Among topoisomerase inhibitors, alterations in *APC* (OR: 1.64, 95% CI: 1.42–1.91; *p* < 0.001), *RB1* (OR: 1.27, 95% CI: 1.03–1.46; *p* = 0.024), and *TP53* (OR: 1.22, 95% CI: 1.03–2.46; *p* = 0.022) were linked to higher ORRs, whereas *PIK3CA* mutations were associated with lower ORRs (OR: 0.75, 95% CI: 0.61–0.91; *p* = 0.004). (Fig. 1e) Overall, these findings indicate that chemotherapy responses varied according to specific gene alterations.

### Associations of genetic mutations and cancer origin on chemotherapy response

To evaluate our hypothesis that chemotherapy response is more influenced by genetic aberrations than the tissue of origin, we developed three predictive models: (1) Model 1, which used significant genes from multivariable analysis; (2) Model 2, which used the primary organ of cancer; and (3) Model 3, which combined both gene and organ information. Each model’s predictive performance was assessed through logistic regression and measured by the AUC, with 5-fold cross-validation used for model validation.

**Fig. 2:**
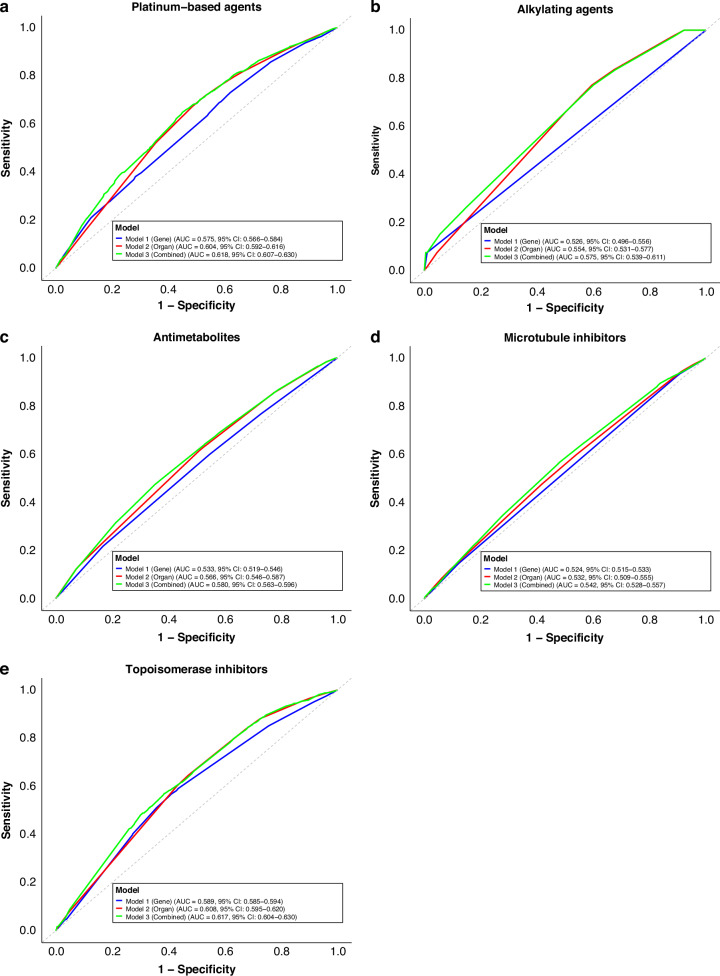
Receiver Operating Characteristic (ROC) curves of overall response rate for each prediction model. ROC curves illustrate the predictive performance of different models across five drug categories: **a** Platinum-based drugs; **b** Alkylating agents; **c** Antimetabolites; **d** Microtubule inhibitors; **e** Topoisomerase inhibitors. Individual ROC curves and the mean ROC curve for each model are shown.

For platinum-based chemotherapy, the gene-only model (Model 1) did not outperform the organ-only model (Model 2). Model 1 had a mean AUC of 0.575 (95% CI: 0.566–0.584), whereas Model 2 showed a mean AUC of 0.604 (95% CI: 0.592–0.616). (Fig. 2a) Notably, combining genetic and organ data (Model 3) achieved the highest performance, with a mean AUC of 0.618 (95% CI: 0.607–0.630). DeLong’s test on pooled data confirmed that Model 3 exhibited significantly better discriminatory power than either Model 1 or Model 2 (*p* < 0.001 for both). Thus, while genomic data alone did not outperform organ-based predictors, integrating both showed improved accuracy. However, the absolute discriminatory ability of all three models was modest, and the AUC values indicate limited clinical utility.

For the other drug classes, Model 2 generally exhibited a higher mean AUC than Model 1, although the difference reached statistical significance only for antimetabolites. (Fig. 2b–e) Model 3 consistently yielded the highest AUC in all drug classes, achieving statistical significance in all but the alkylating agent group. Taken together, these findings do not support the hypothesis that genetic information alone is more predictive of chemotherapy response than organ of origin. Detailed AUC values are presented in Supplementary Table 3 and DeLong’s test results are presented in Supplementary Table 4. However, as with platinum-based chemotherapy, the discriminatory ability across all models remained modest, underscoring their limited clinical utility. The timing of treatment (first-line vs second-line or later) did not change the result (data not shown).

Since CGP testing typically identifies multiple variants, distinguishing between driver and passenger mutations is often challenging. To explore the potential impact of variant composition, we conducted a subgroup analysis comparing tumors with high tumor mutation burden (>10 mutations/Mb), which are likely to include a higher proportion of passenger mutations, with tumors with low TMB ( < 5 mutations/Mb), in which driver mutations are expected to represent a larger fraction. The results of this analysis are shown in Supplementary Figure 3. Across all drug classes, Model 3 consistently showed the highest AUC in the TMB-low group. In contrast, the TMB-high group exhibited a different trend, suggesting that an increased proportion of passenger mutations may compromise predictive performance.

### Gene-organ interactions and time to next treatment analysis

In oncology, not only response rates but also the duration of treatment benefit are critical. We therefore analyzed TNT to assess how long regimens remained effective. Initially, we conducted a pan-cancer analysis and then performed organ- and gene-specific analyses to explore gene-organ-drug interactions.

**Fig. 3:**
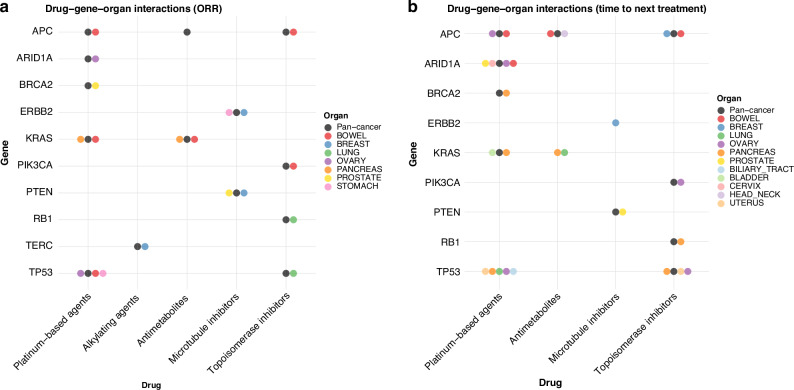
Drug-gene-organ interactions and their association with overall response rate and time to next treatment (TNT). This figure presents the relationship between specific gene alterations, primary cancer organs, and response to chemotherapy, as well as their impact on TNT. Each circle represents the drug-gene-organ combination that yielded statistically significant difference (*p* < 0.05) compared to wild type.

Figure 3a highlights significant differences in ORRs by gene-organ-drug combination (*p* < 0.05 on odds ratio). Figure 3b illustrates significant variations in TNT by gene alteration within specific organs (*p* < 0.05 on hazard ratio). Supplementary Fig. 2 provides further details, including forest plots of HR of TNT and ORR analyses stratified by gene status, organ, and drug class.

### Platinum-based drugs

In the pan-cancer analysis, patients with *APC* mutation showed significantly longer TNT for platinum-containing regimens (HR = 0.68, 95% CI: 0.64–0.71; *p* < 0.001). *BRCA2* (HR = 0.88, 95% CI: 0.82–0.94; *p* < 0.001) and *KRAS* (HR = 0.89, 95% CI: 0.85–0.94; *p* < 0.001) mutations were also associated with longer TNT, whereas *ARID1A* mutations correlated with shorter TNT (HR = 1.22, 95% CI: 1.14–1.31; *p* < 0.001) (Fig. 3b).

In colorectal cancer, *APC* mutations were associated with significantly longer TNT (HR = 0.82, 95% CI: 0.73–0.92; *p* < 0.001), suggesting enhanced platinum sensitivity in *APC*-mutated tumors. (Fig. 3b and Supplementary Fig. 2A) Previous small-scale studies (1–32 cases) have reported contradictory results for *APC* mutation and oxaliplatin-containing regimens [15–17]. However, our larger sample (*n* = 1881) showed the opposite trend, underscoring the need for further validation. In pancreatic cancer, *KRAS* mutations correlated with shorter TNT (HR = 1.33, 95% CI: 1.12–1.58; *p* < 0.001), implying reduced responsiveness to platinum regimens. *KRAS* mutations are highly prevalent in pancreatic cancer [18, 19], limiting comparisons with wild-type cases. However, the overall trend in TNT analysis for *KRAS* alteration showed different patterns depending on the organ types. Of particular interest, *BRCA2* mutation was associated with higher response rates and prolonged TNT for platinum-based regimens in multiple tumor types, including pancreatic and breast cancers (Supplementary Fig. 2A), extending prior reports in breast, ovarian, and pancreatic malignancies [20–26]. By contrast, no similar benefit was observed for other homologous recombination repair genes, such as *PALB2* or *RAD51* (data not shown).

### Alkylating agents

Most alkylating agent regimens were administered to patients with breast cancer. Among those harboring TERC mutations, cyclophosphamide-containing regimens yielded a significantly higher ORR (75.0% vs. 18.3%; *p* < 0.001) and a trend toward longer TNT (HR = 0.64, 95% CI: 0.31–1.29; *p* = 0.208). (Supplementary Fig. 2B) Prior studies have suggested that telomerase activation (through high TERT expression or gene amplification) might reduce response to cyclophosphamide [27, 28], but our data suggest the opposite for TERC mutations, warranting further investigation.

### Antimetabolites

In the pan-cancer analysis, *APC*-mutated tumors exhibited longer TNT (HR = 0.80, 95% CI: 0.77–0.83; *p* < 0.001) for antimetabolite regimens. (Fig. 3b) However, *KRAS* mutations did not show a statistically significant difference in TNT (HR = 0.98, 95% CI: 0.94–1.02; *p* = 0.272).

In colorectal cancer, *APC* mutations did not significantly increase ORR for antimetabolite-based treatments, but TNT was prolonged (HR = 0.91, 95% CI: 0.84–0.99; *p* = 0.040). (Fig. 3a, b, Supplementary Fig. 2C) Antimetabolite therapy in colorectal cancer is often combined with platinum or topoisomerase inhibitors, and additional analyses revealed that platinum and topoisomerase inhibitors, rather than antimetabolites alone, primarily drove longer TNT (Table 2).

**Table 2:** Gene mutation status and treatment efficacy comparison across different cancer therapies.

Organ—Gene	Treatment	*N*	Hazard Ratio of TNT	95% CI	*P*_value
Colorectal Cancer—APC	Antimetabolites (+) & Platinum-based drugs (+)	1250	0.84	[0.73, 0.97]	0.015
	Antimetabolites (+) & Platinum-based drugs (-)	2574	0.93	[0.84, 1.05]	0.241
	Antimetabolites (-) & Platinum-based drugs (+)	643	0.76	[0.63, 0.94]	0.009
Colorectal Cancer—APC	Antimetabolites (+) & Topoisomerase inhibitors (+)	1399	0.86	[0.74, 0.99]	0.037
	Antimetabolites (+) & Topoisomerase inhibitors (-)	2425	0.94	[0.85, 1.06]	0.311
	Antimetabolites (-) & Topoisomerase inhibitors (+)	710	0.74	[0.6, 0.91]	0.004
Colorectal Cancer—KRAS	Antimetabolites (+) & Platinum-based drugs (+)	1250	1.06	[0.95, 1.18]	0.32
	Antimetabolites (+) & Platinum-based drugs (-)	2574	1.04	[0.96, 1.12]	0.336
	Antimetabolites (-) & Platinum-based drugs (+)	643	1.02	[0.87, 1.19]	0.804
Pancreas Cancer—KRAS	Antimetabolites (+) & Platinum-based drugs (+)	741	1.23	[1.02, 1.49]	0.032
	Antimetabolites (+) & Platinum-based drugs (-)	1950	1.19	[1.06, 1.33]	0.003
	Antimetabolites (-) & Platinum-based drugs (+)	158	1.61	[1.09, 2.36]	0.016
Breast Cancer—TERC	Adriamycin & Cyclophosphamide	56	0.67	[0.16, 2.76]	0.575
	Epirubicin & Cyclophosphamide	104	0.74	[0.27, 2.03]	0.565
	Other regimen including Cyclophosphamide	66	0.52	[0.13, 2.13]	0.363
Breast Cancer—ERBB2	Vinorelbine	142	0.62	[0.42, 0.93]	0.02
	Eribulin	626	0.85	[0.7, 1.05]	0.128
	Taxanes	613	0.99	[0.81, 1.21]	0.907
Prostate Cancer—PTEN	Cabazitaxel	187	1.54	[1.03, 2.31]	0.037
	Docetaxel	511	1.19	[0.93, 1.52]	0.158

In pancreatic cancer with *KRAS* mutation, ORR was low, and TNT was significantly shorter (HR = 1.20, 95% CI: 1.09–1.33; p < 0.001) for antimetabolite-containing regimens. (Fig. 3a, b, Supplementary Figure 2C) Combination chemotherapy regimens in pancreatic cancer often include platinum-based drugs plus antimetabolites. Our additional analysis indicated that *KRAS*-altered pancreatic cancer patients showed worse TNT regardless of the specific cytotoxic drug combination, suggesting broader chemoresistance conferred by *KRAS* (Table 2).

### Microtubule inhibitors

In the pan-cancer analysis, *ERBB2* (HER2) alterations did not significantly affect TNT (HR = 0.98, 95% CI: 0.91–1.05; *p* = 0.544), whereas *PTEN* mutations were associated with shorter TNT (HR = 1.15, 95% CI: 1.06–1.25; *p* < 0.001).

Among breast cancers harboring *ERBB2* alteration, ORR was higher for microtubule inhibitors (28.2% vs. 21.7%; p = 0.014), with a corresponding prolongation in TNT (HR = 0.82, 95% CI: 0.73–0.92; *p* = 0.001). (Supplementary Fig. 2D) Many patients received concurrent anti-HER2 therapy (e.g., trastuzumab) with taxanes, a combination known for synergy [29–31]. Interestingly, other microtubule inhibitors (e.g., eribulin, vinorelbine) also showed a trend toward better TNT in *ERBB2*-altered breast cancer; vinorelbine, in particular, reached statistical significance (HR = 0.62, 95% CI: 0.43–0.91; *p* = 0.013) (Table 2), although further research is needed [32, 33]. In prostate cancer, *PTEN* loss correlated with shorter TNT (HR = 1.27, 95% CI: 1.03–1.57; *p* = 0.024), consistent with earlier studies demonstrating taxane resistance in *PTEN*-altered disease [34, 35].

### Topoisomerase Inhibitors

In the pan-cancer analysis, *APC* alterations were strongly linked to longer TNT (HR = 0.58, 95% CI: 0.55–0.61; *p* < 0.001), and *TP53* mutations also correlated with prolonged TNT (HR = 0.84, 95% CI: 0.79–0.89; *p* < 0.001). (Fig. 3b) Conversely, *PIK3CA* (HR = 1.10, 95% CI: 1.02–1.19; *p* = 0.013) and *RB1* (HR = 1.10, 95% CI: 1.01–1.21; *p* = 0.023) mutations were associated with shorter TNT.

In colorectal cancer, *APC* mutations conferred a higher ORR (27.6% vs. 19.2%; *p* = 0.002) and longer TNT (HR = 0.83, 95% CI: 0.73–0.93; *p* = 0.001). (Supplementary Fig. 2E) As with platinum-based and antimetabolite regimens, the strongest signal for *APC*-associated benefit appeared to involve platinum and topoisomerase inhibitors rather than antimetabolites alone (Table 2).

In pancreatic cancer, *RB1* mutations were linked to shorter TNT (HR = 1.48, 95% CI: 1.04–2.10; *p* = 0.030), and *TP53* mutations similarly correlated with worse survival (HR = 1.23, 95% CI: 1.07–1.42; *p* = 0.004). These findings align with earlier work suggesting that *RB1* loss in pancreatic cancer, particularly in pancreatic neuroendocrine carcinoma, confers platinum–etoposide sensitivity, whereas *TP53* mutations in pancreatic ductal adenocarcinoma predict poorer outcomes with FOLFIRINOX regimen [36, 37].

## Discussion

Recent advances in cancer genomics have enabled the development of molecularly targeted therapies. Tumor-agnostic approval of molecularly targeted agents is on the rise, sparking discussions about reclassifying tumors by molecular pathways rather than their anatomical origin. Although the links between genomic alterations and efficacy of targeted therapies are well characterized, the relationship between genomic alterations and chemotherapy response remains underexplored.

Our study set out to examine the long-standing assumption that chemotherapy response was primarily determined by organ types. We hypothesized that genomic aberrations, reflecting cellular functions, might provide stronger predictive value. Leveraging a large real-world dataset of 15,474 patients from the C-CAT database, our results did not support this hypothesis. In most cases, organ-based predictions outperformed gene-only models, although combining genetic and organ-specific factors provided the best performance.

Mechanistically, most cytotoxic chemotherapies target fundamental processes such as DNA replication, mitotic spindle assembly, or microtubule dynamics, pathways that are broadly conserved across tumor types rather than being driven by specific oncogenic mutations. While pathway-targeted therapies (e.g., kinase inhibitors) have transformed treatment in select genomic subgroups, our results suggest that fundamental cellular functions such as mitotic control remain the primary determinants of response to “classical” chemotherapies. This may explain why allele-specific aberrations failed to recapitulate tissue-level effects in our predictive models.

Several gene-organ associations were associated with improved responses. *APC* mutations appeared to be associated with prolonged TNT across multiple drug classes—especially platinum-based and topoisomerase inhibitor regimens—in colorectal cancer. Conversely, *KRAS* mutations were observed to correlate with poorer outcomes, particularly in pancreatic cancer. *BRCA2* alterations were linked to increased sensitivity to platinum-based agents across various malignancies, broadening evidence from breast, ovarian, and pancreatic cancers to additional tumor types.

These exploratory findings suggest that combining organ-based and molecular data may warrant further investigation as an approach to refine chemotherapy response prediction, though clinical applicability remains uncertain.

Leveraging a nation-wide, real-world dataset of over 15,000 patients, this study provides rare and valuable insights into gene–drug–organ associations in the context of standard chemotherapy. While previous studies have examined specific gene–drug relationships, they were often limited to small cohorts or single tumor types. In contrast, our pan-cancer analysis, based on insurance-covered clinical data, reflects actual oncology practice across a broad spectrum of malignancies in Japan. Moreover, unlike prior work focusing on molecularly targeted therapies, we addressed the underexplored question of whether responses to conventional chemotherapy are primarily driven by genomic alterations, organ-specific factors, or their combination. This exploratory approach contributes important real-world evidence and may offer a new perspective on the personalization of cytotoxic chemotherapy.

Despite these insights, our study had several limitations. First, this was a retrospective analysis, and chemotherapy was administered only to cancer types for which efficacy had already been validated. Therefore, the observed associations may not reflect direct predictive effects of specific variants, but rather underlying tumor-type–specific treatment strategies. This introduces a strong bias toward demonstrating effectiveness in certain organ types. In addition, potential confounding may have arisen from the non-randomized association between specific drug classes and specific tumor types and sample sizes, which is inherent in real-world clinical practice. Given the impracticality of prospectively administering chemotherapy across all organ types, our study currently represents the best available evidence. Second, treatment decisions were not randomized but guided by real-world clinical practices, resulting in heterogeneous treatment approaches. Third, certain influential factors, such as comorbidities, were not captured in the dataset. Fourth, our analysis did not explicitly distinguish clonal hematopoiesis of indeterminate potential (CHIP) from bona fide tumor-derived pathogenic variants. Therefore, the interpretation of variants potentially associated with CHIP—particularly in liquid biopsy samples—should be approached with caution. Lastly, some findings—particularly those related to rare mutations such as *TERC*—require validation in larger cohorts.

In this nationwide real-world analysis, genomic aberrations alone did not outperform tumor organ type in predicting chemotherapy response. Although integrating genomic and organ-specific data yielded statistically higher AUCs, the discriminatory ability remained modest and of limited clinical utility. These results should be interpreted as exploratory and hypothesis-generating. Larger prospective studies are required to validate these exploratory associations before considering any application to personalized chemotherapy.

## Supplementary information

SUPPLEMENTARY TABLES AND FIGURES

## Data Availability

The data that support the findings of this study are available from the corresponding author upon reasonable request.
