# Correction: Exploratory analysis of gene aberrations and chemotherapy response: findings from a real-world database in Japan

**DOI:** 10.1038/s41416-026-03377-2

**Published:** 2026-03-30

**Authors:** Naoya Ishibashi, Takashi Kamatani, Satoru Aoyama, Masanori Tokunaga, Yusuke Kinugasa, Sadakatsu Ikeda

**Affiliations:** 1 Department of Precision Cancer Medicine, Institute of Science Tokyo Hospital, Tokyo, Japan; 2 Department of Gastrointestinal Surgery, Institute of Science Tokyo, Tokyo, Japan; 3 Department of AI Technology Development, M&D Data Science Center, Institute of Integrated Research, Institute of Science Tokyo, Tokyo, Japan

**Keywords:** Targeted therapies, Chemotherapy

Correction to: *British Journal of Cancer* 10.1038/s41416-025-03281-1, published online 22 December 2025

In the originally published version of this Article, the images for Figure 1c and Figure 1d were inadvertently duplicated from Figure 1b. This has now been corrected and the accurate Figure 1c and Figure 1d are shown in the updated Figure 1.

The article has been corrected.

Original Figure 1
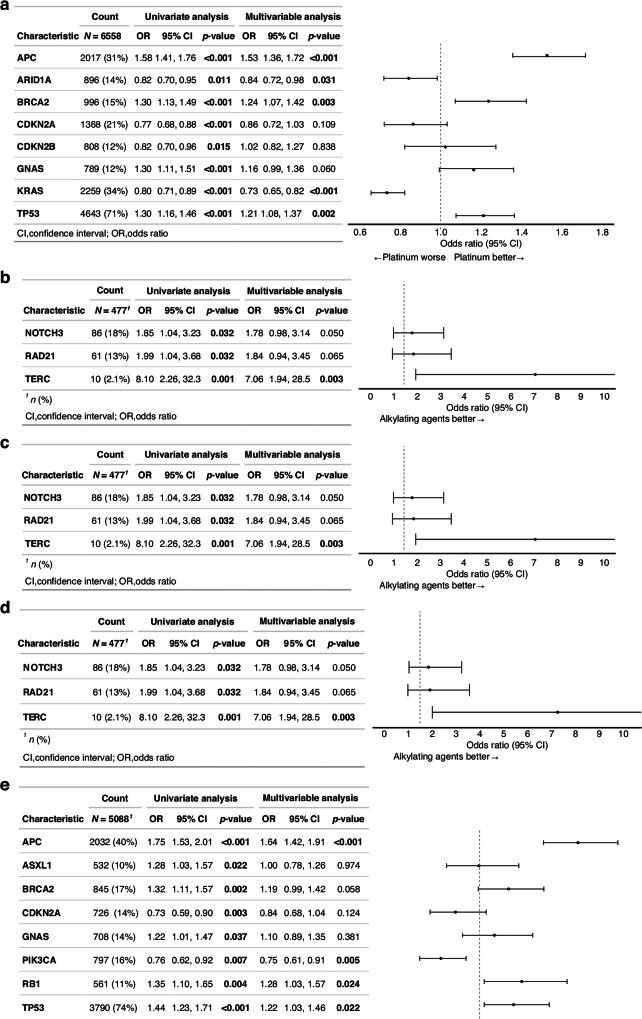


Corrected Figure 1
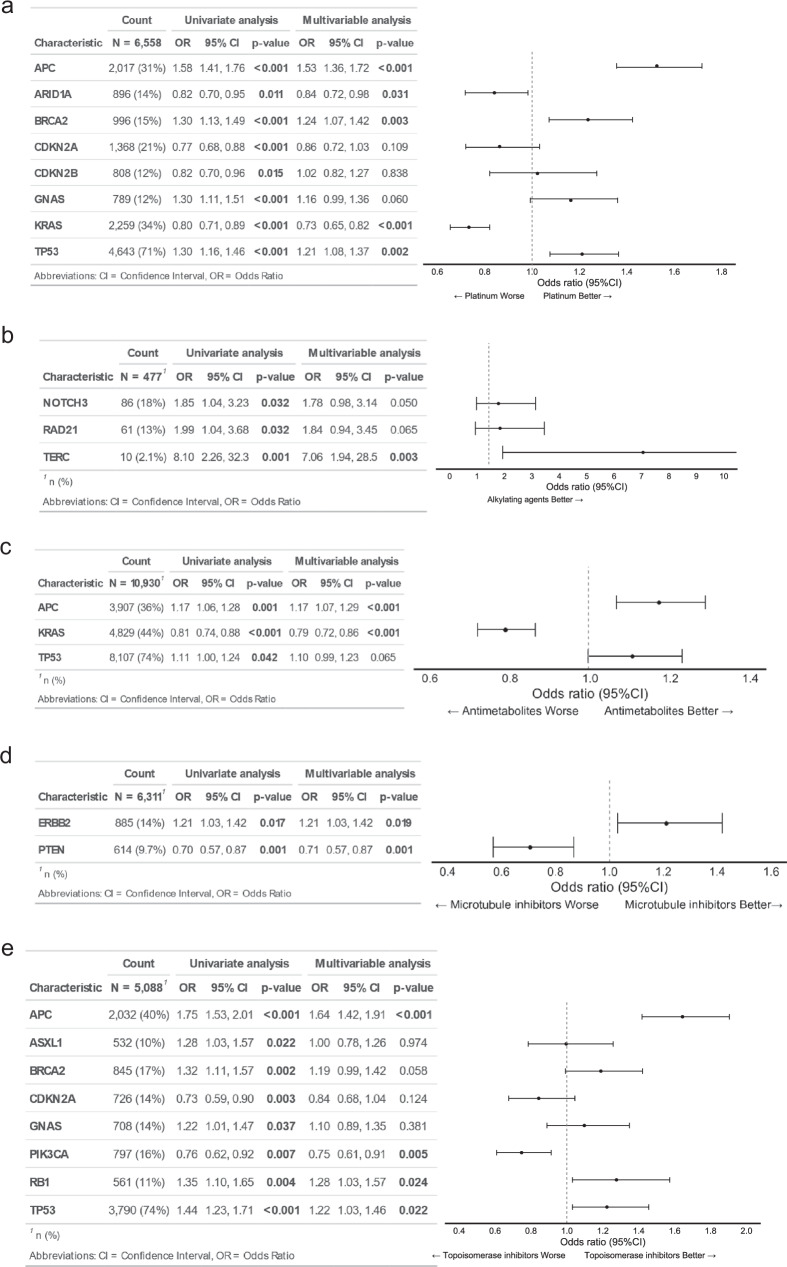


**Figure 1. Multivariable analysis of overall response rate association between chemotherapy response and gene alterations**. The analysis was conducted for five drug categories: **a** Platinum-based drugs, **b** Alkylating agents, **c** Antimetabolites, **d** Microtubule inhibitors, **e** Topoisomerase inhibitors.

The original article has been corrected.

