# Supplementary material for: Exploratory analysis of gene aberrations and chemotherapy response: findings from a real-world database in Japan
Source: Br J Cancer. 2025 Dec 22;134(5):811–9. doi: 10.1038/s41416-025-03281-1 (PMC12905273; doi:10.1038/s41416-025-03281-1)
Supplement: Supplementary file 1 — SUPPLEMENTARY TABLES AND FIGURES [file 41416_2025_3281_MOESM1_ESM.docx]

**SUPPLEMENTARY TABLES AND FIGURES**

**Supplementary Table1: 5 Categories of Chemotherapy Drug Class and Cancers Included in Analysis**

**Supplementary Table2: Full list of genes Included in Comprehensive Genomic Profiling Testing**

**Supplementary Table3: Area under the curve (AUC) Discriminative Performance of 3 Models for Drug Response Across Different Drug Classes**

**Supplementary Table4: Comparison of AUCs Between Gene, Organ, and Combined Models Using Delong's Test Across Different Drug Classes**

**Supplementary Figure 1. Flow diagram of analysis.**

**
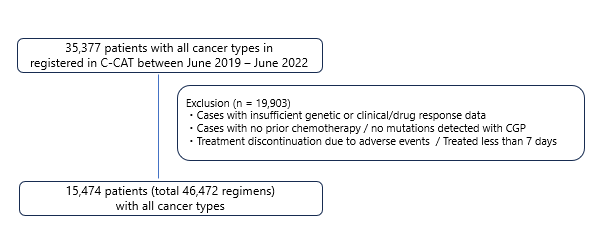
**

**Supplementary Figure 2. Results of the univariate analysis of the ORR for each gene and drug by organ (Left) and the forest plot of the TNT for each organ (Right). A, Platinum-based drugs; B, Alkylating agents; C, Antimetabolites; D, Microtubule inhibitors; E, Topoisomerase inhibitors.**


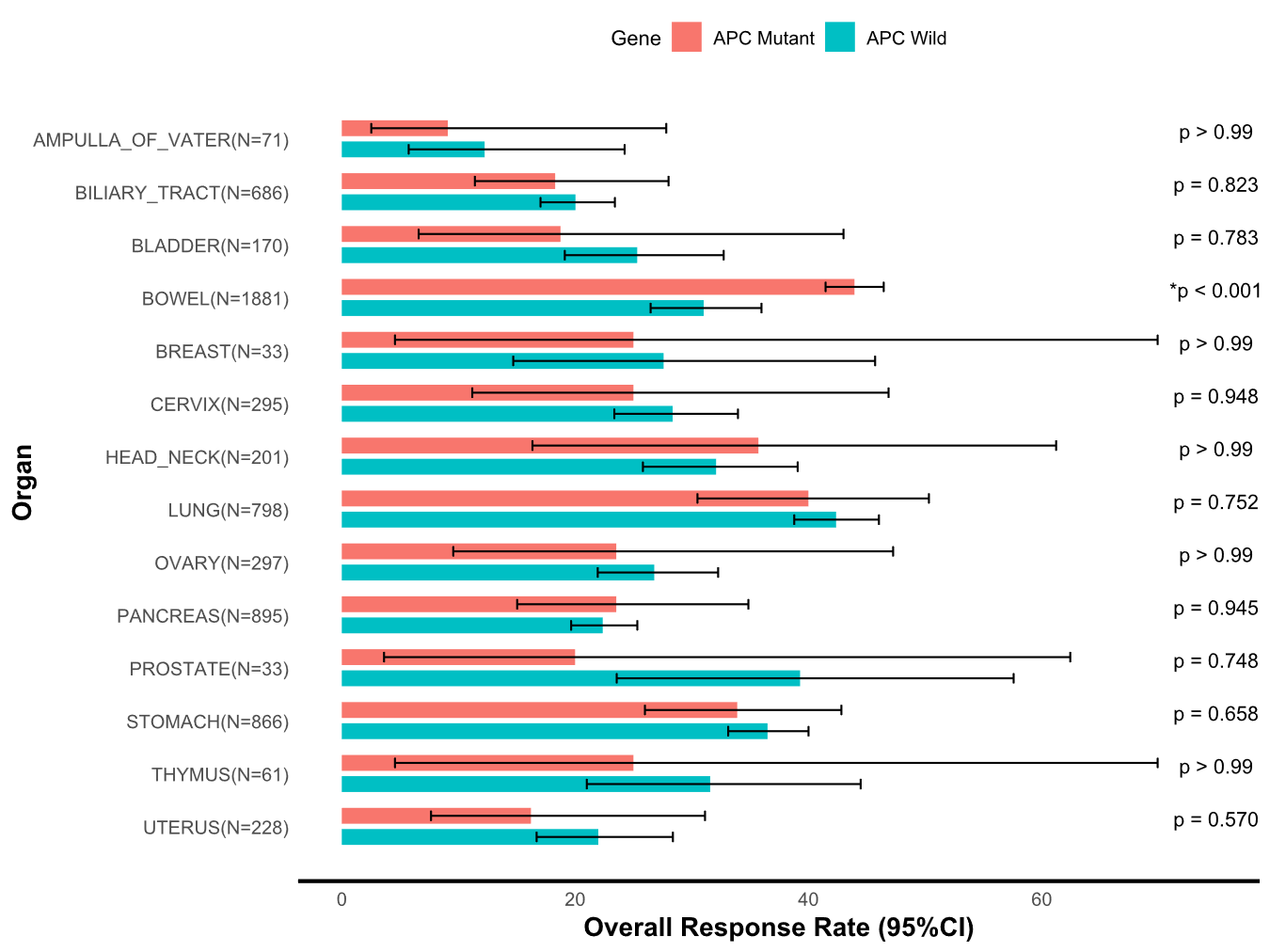
(A)


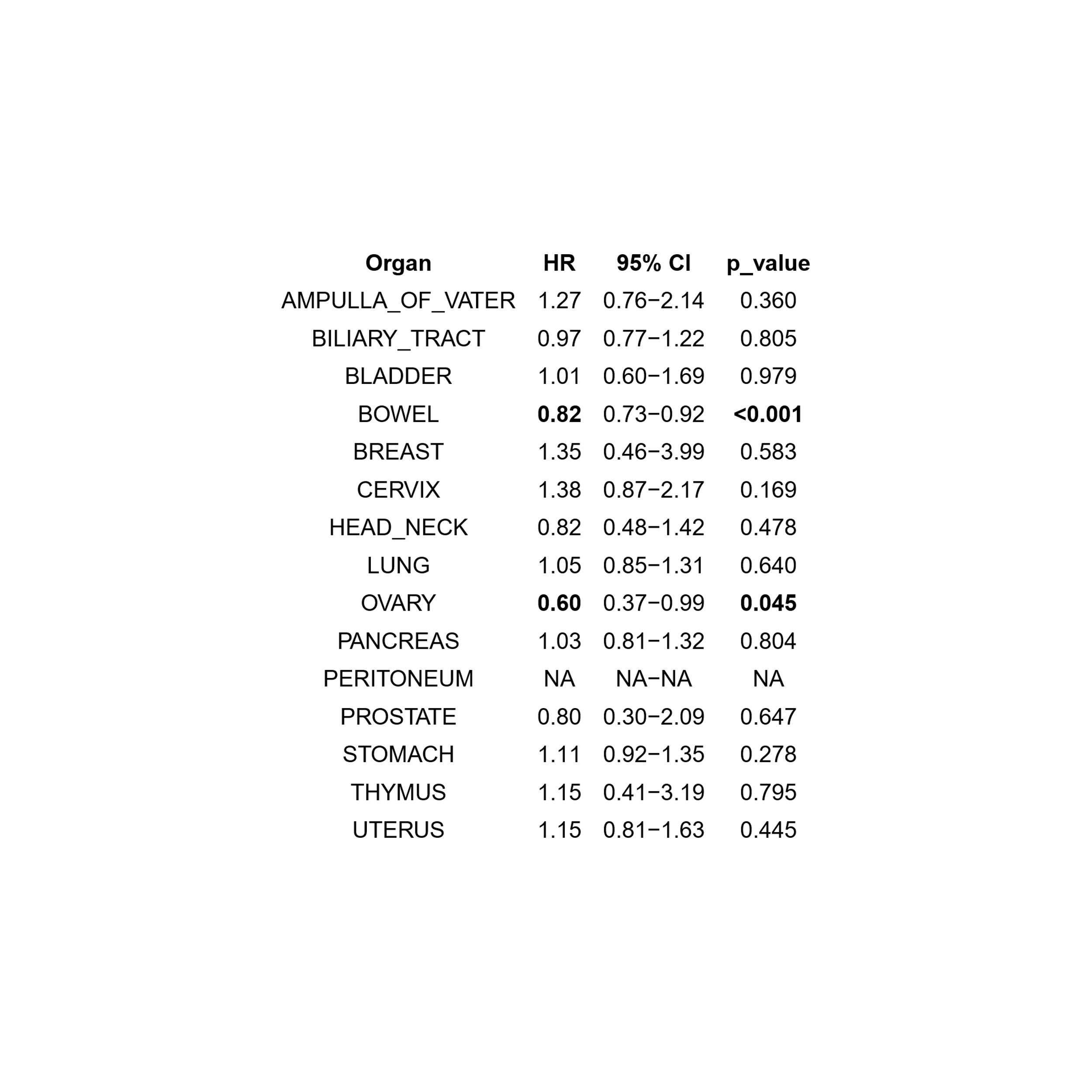

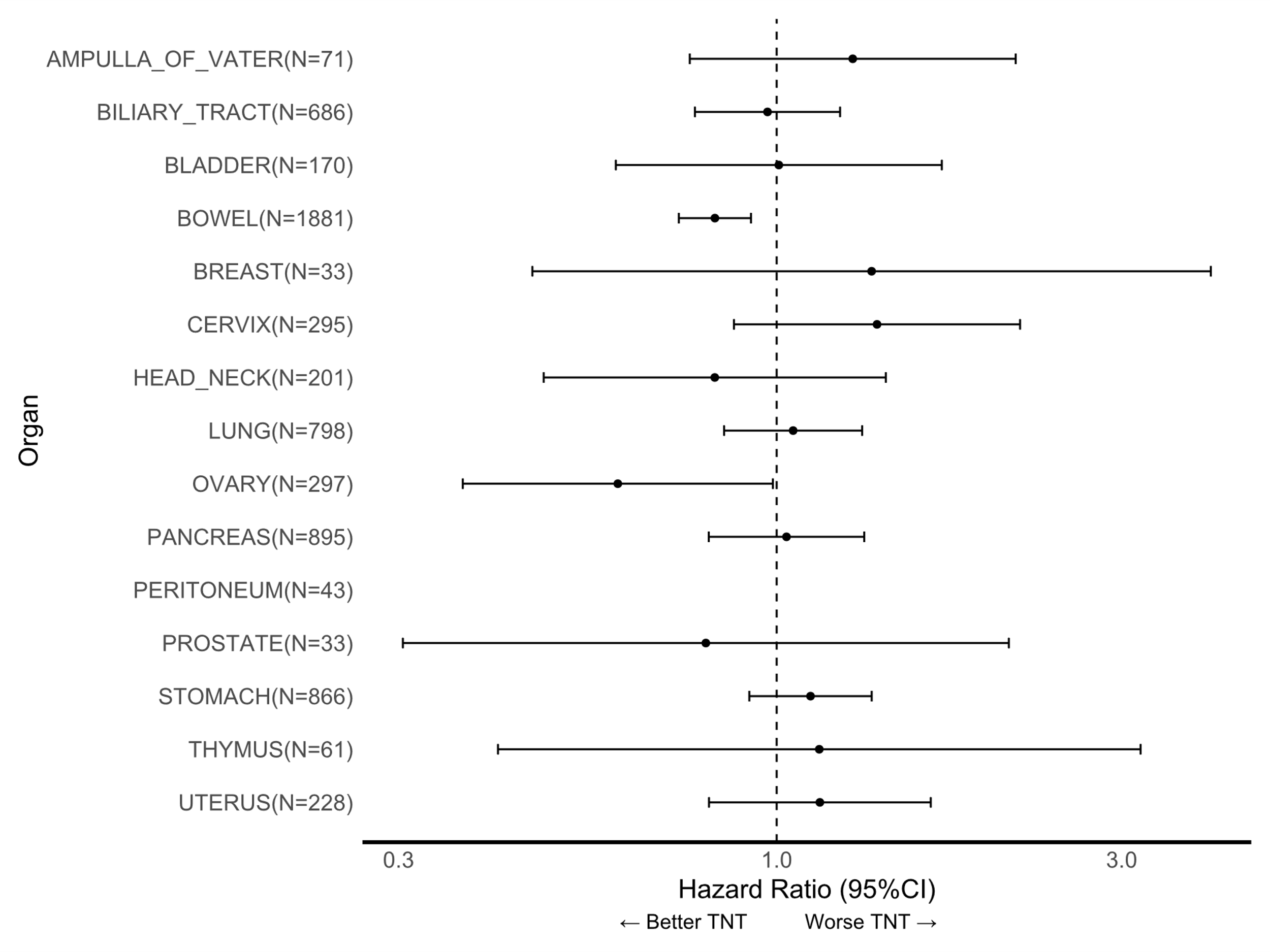


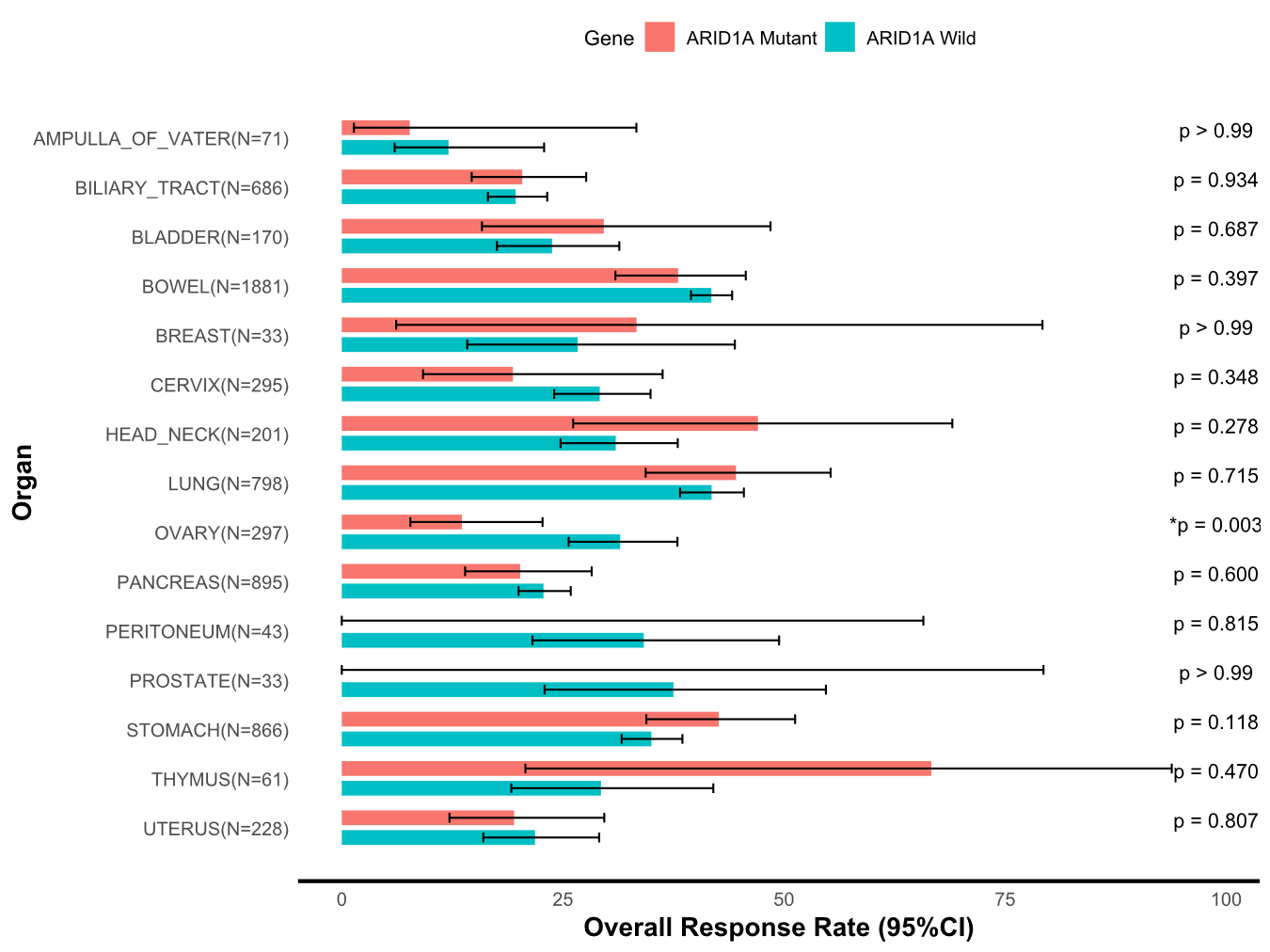


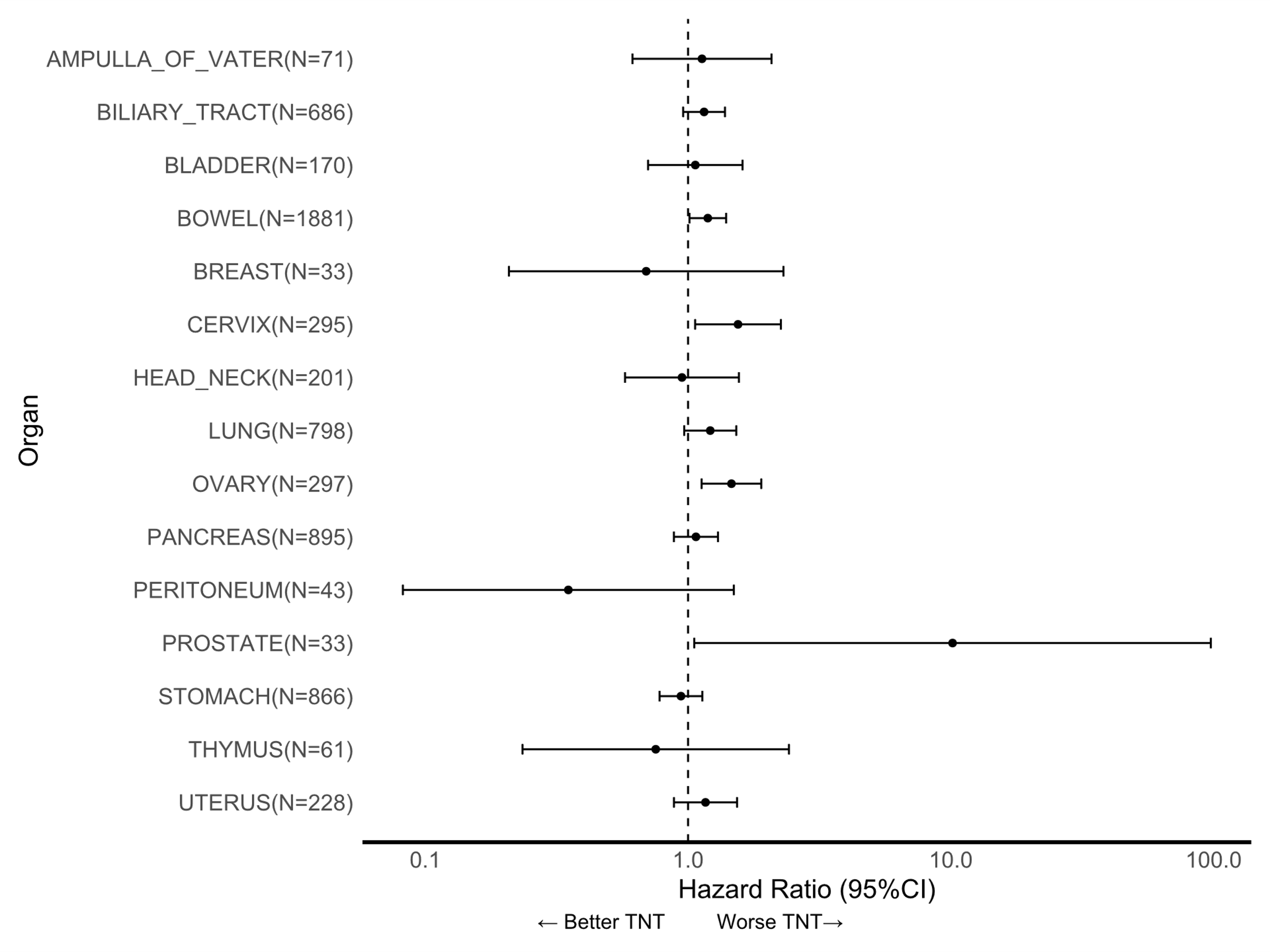

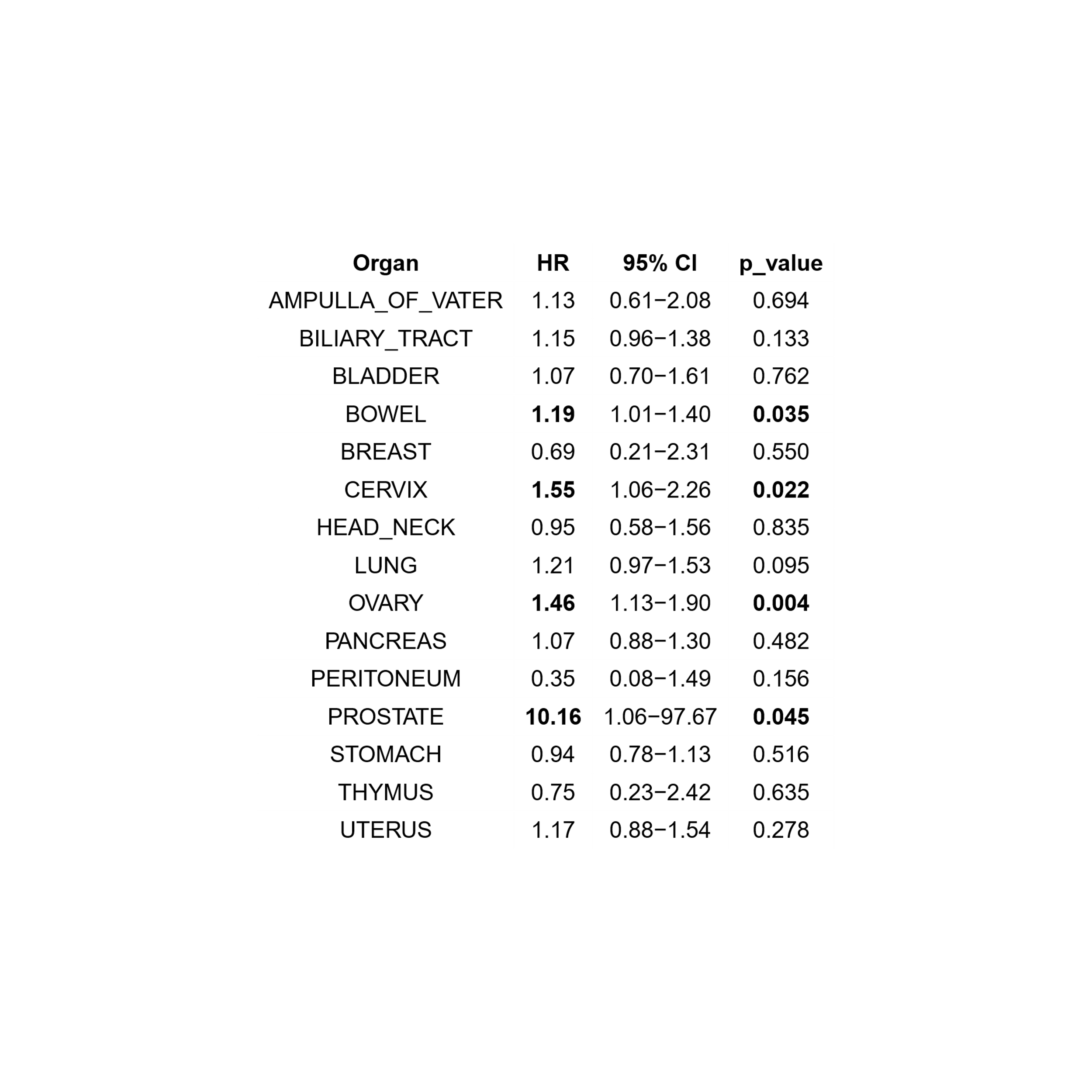


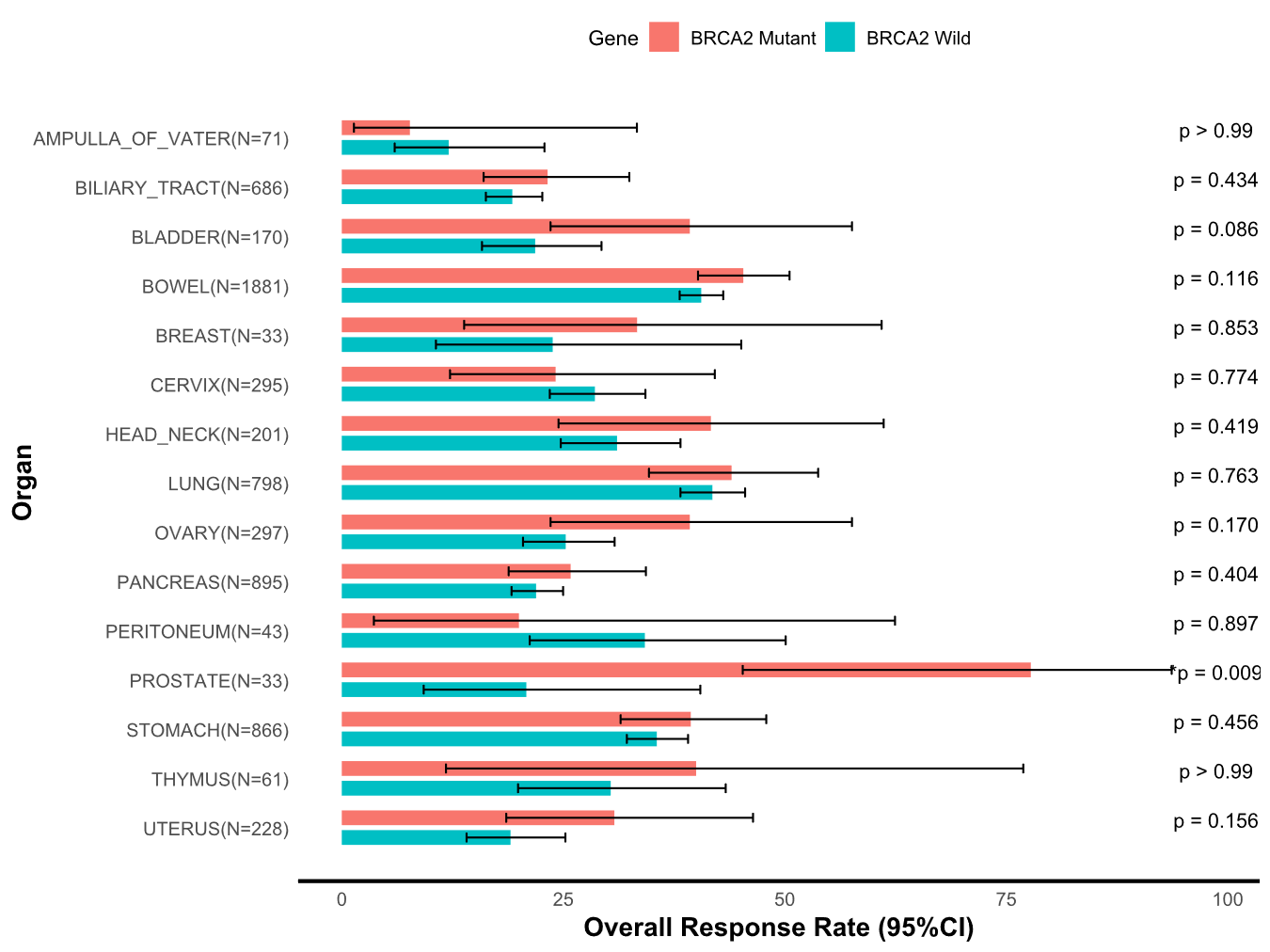


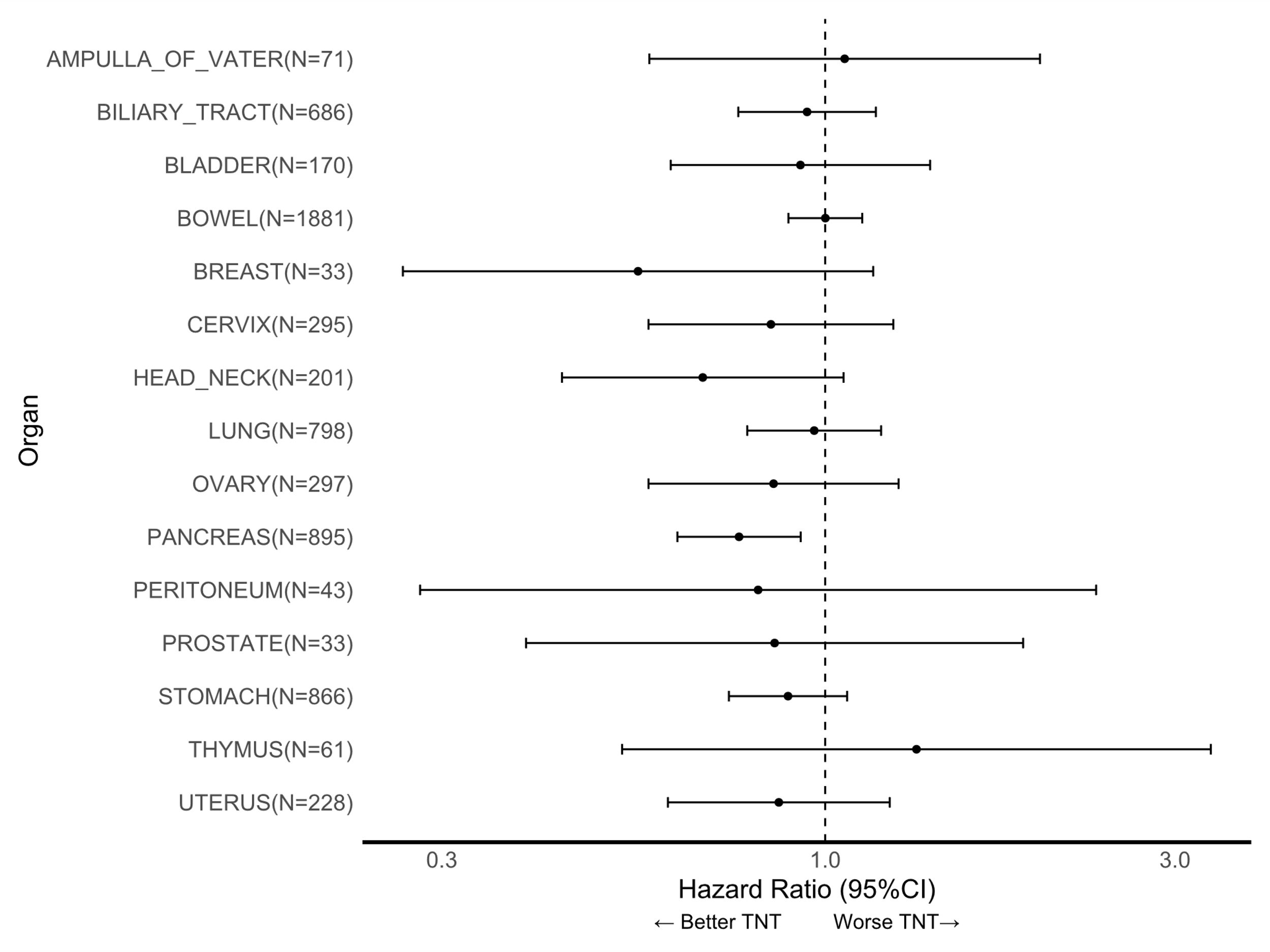

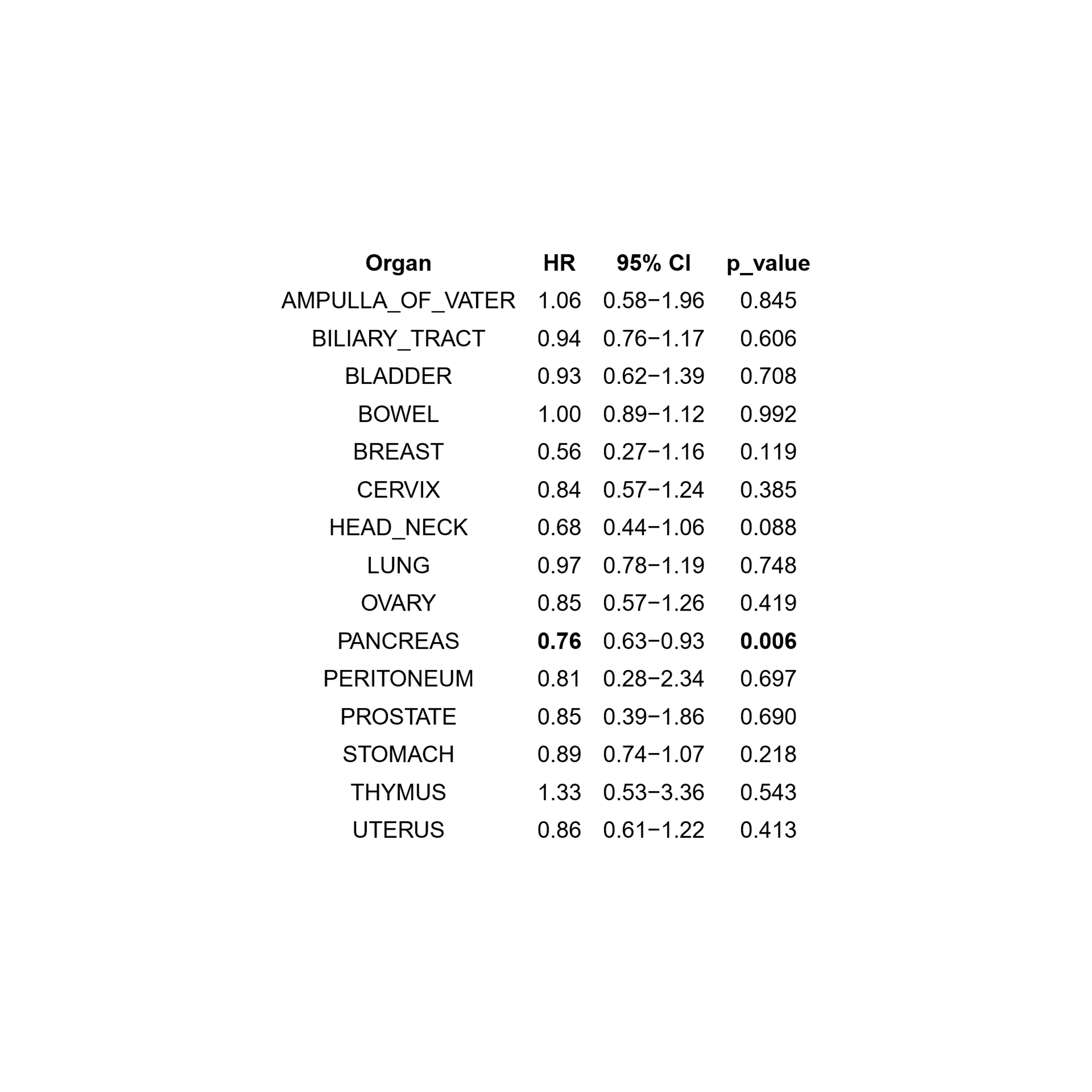


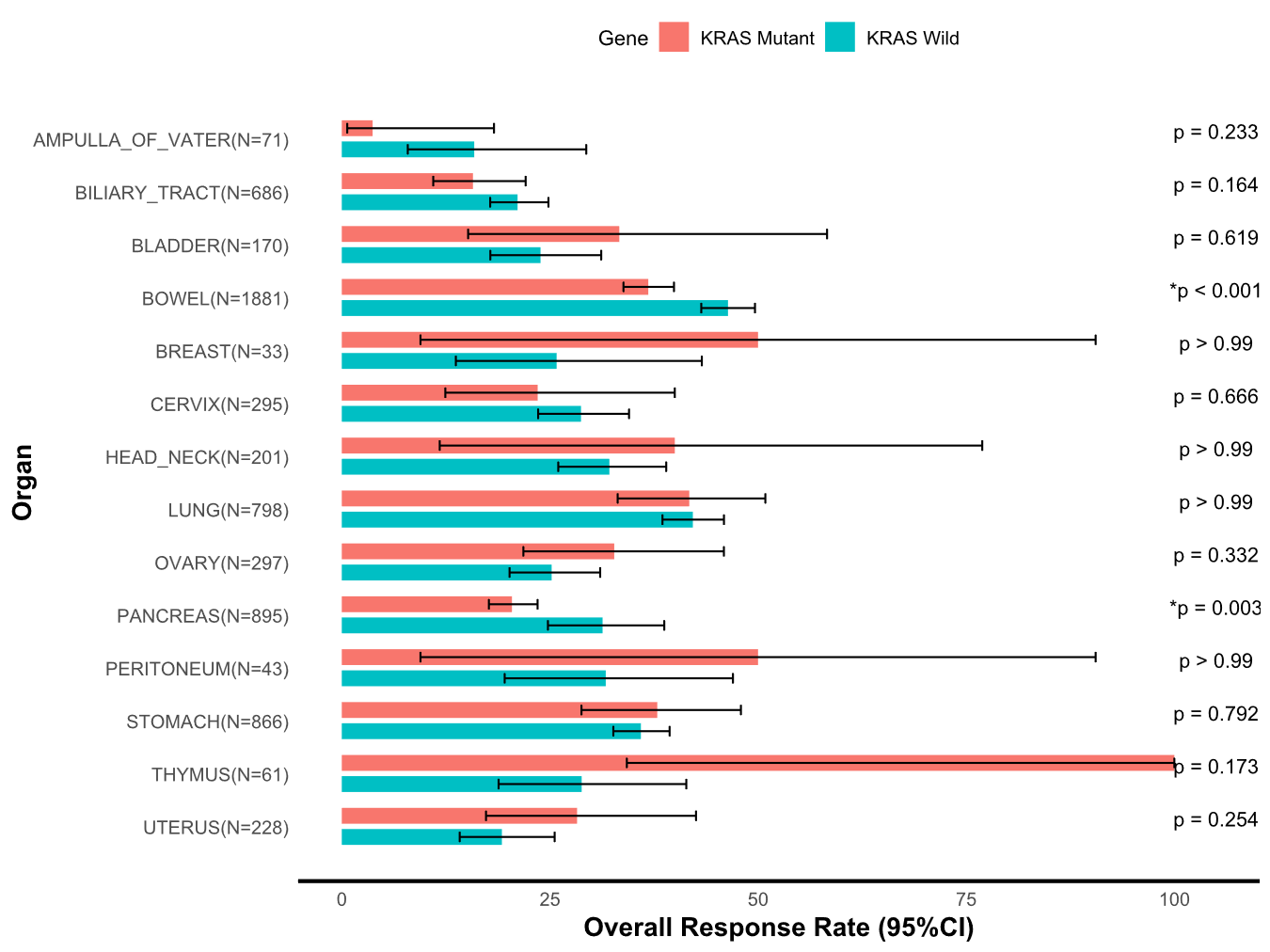


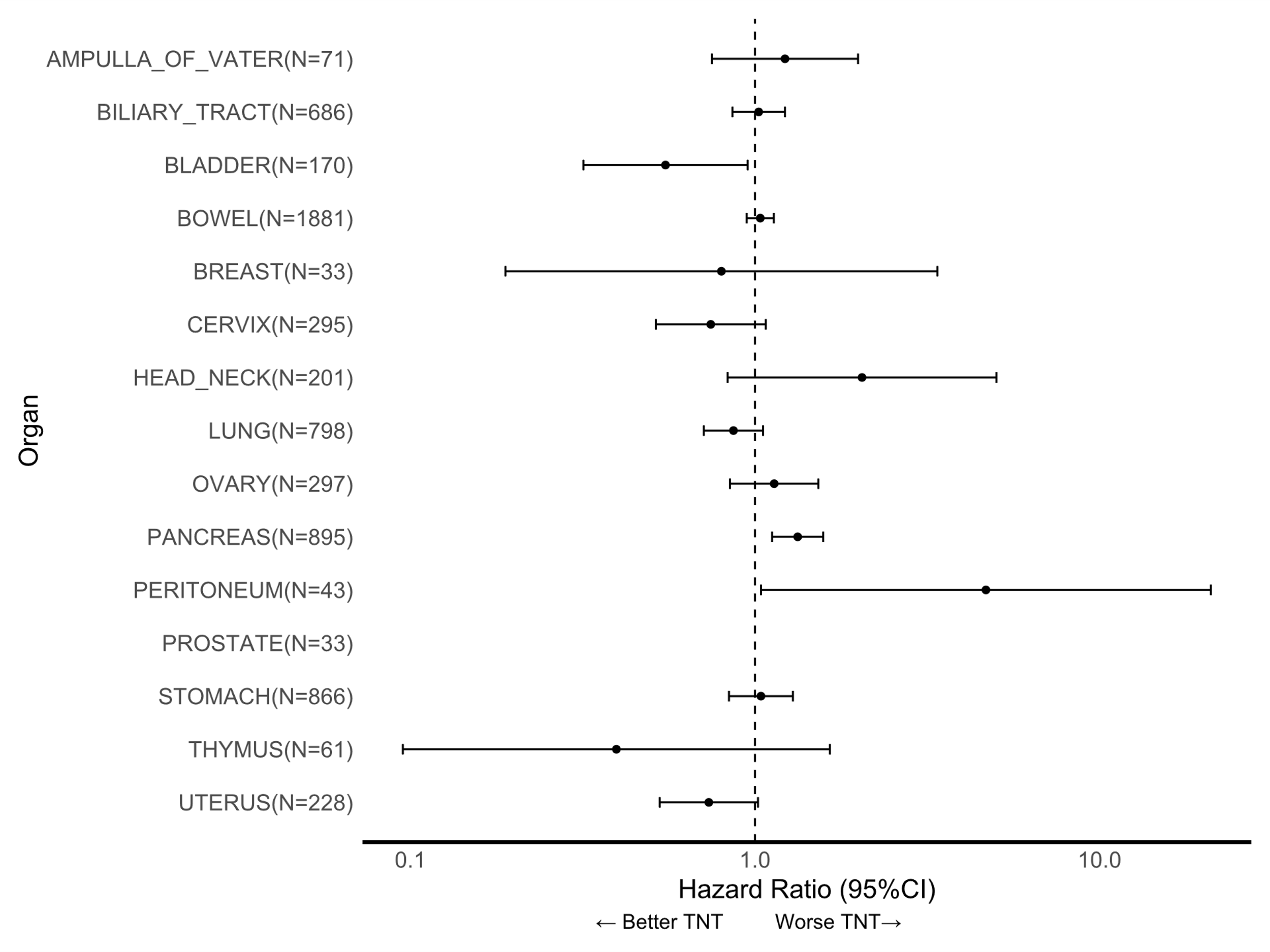

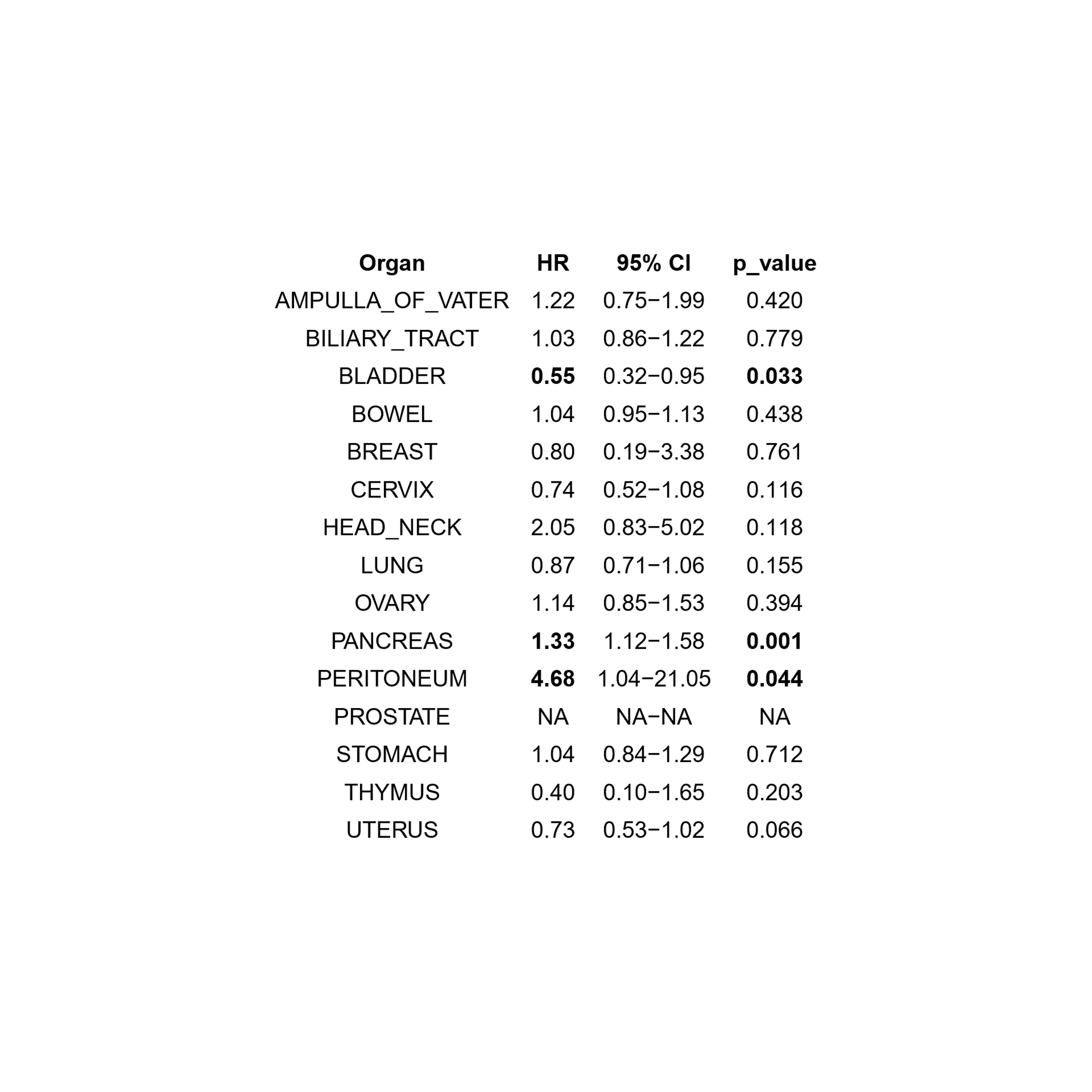


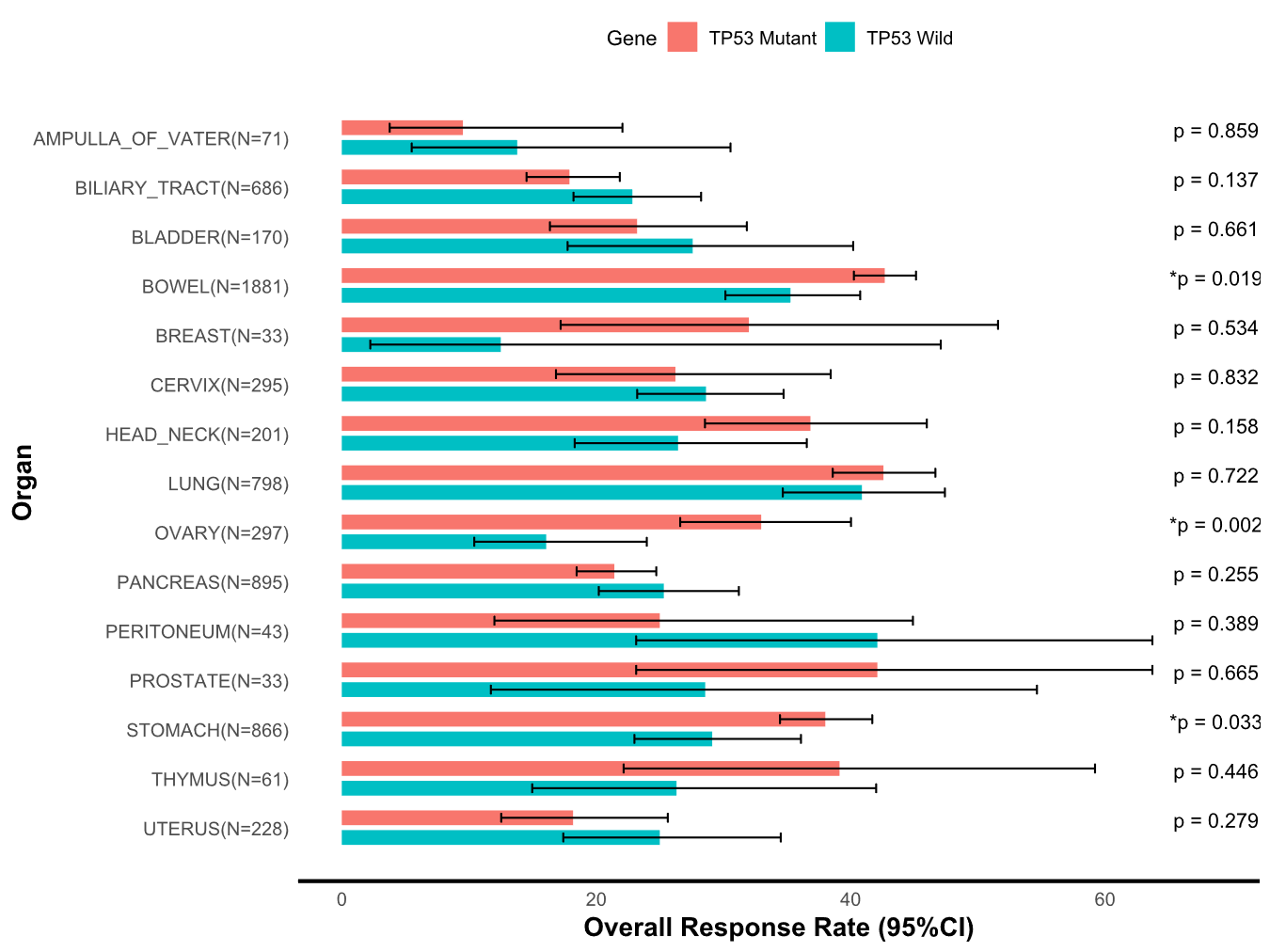


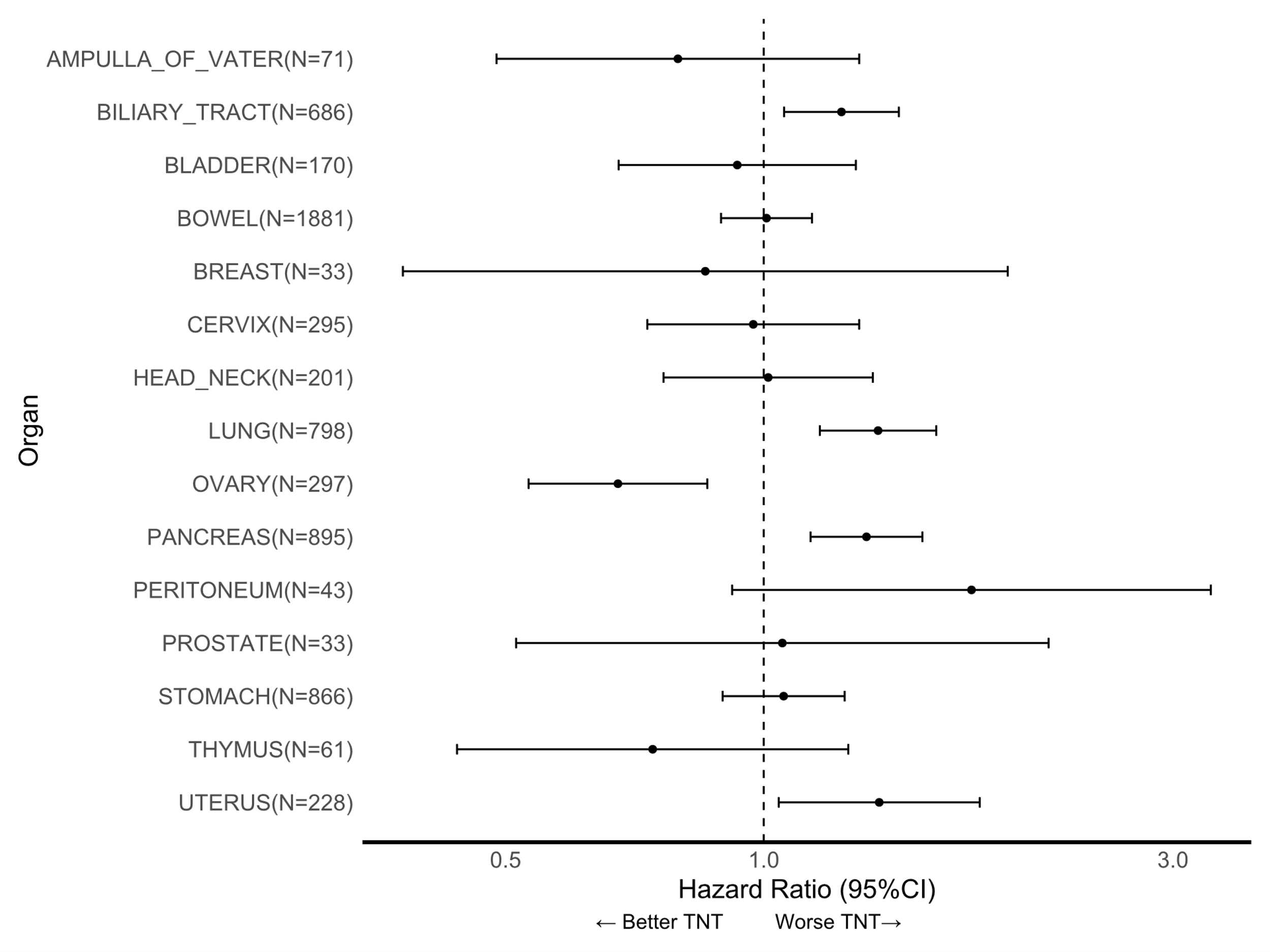

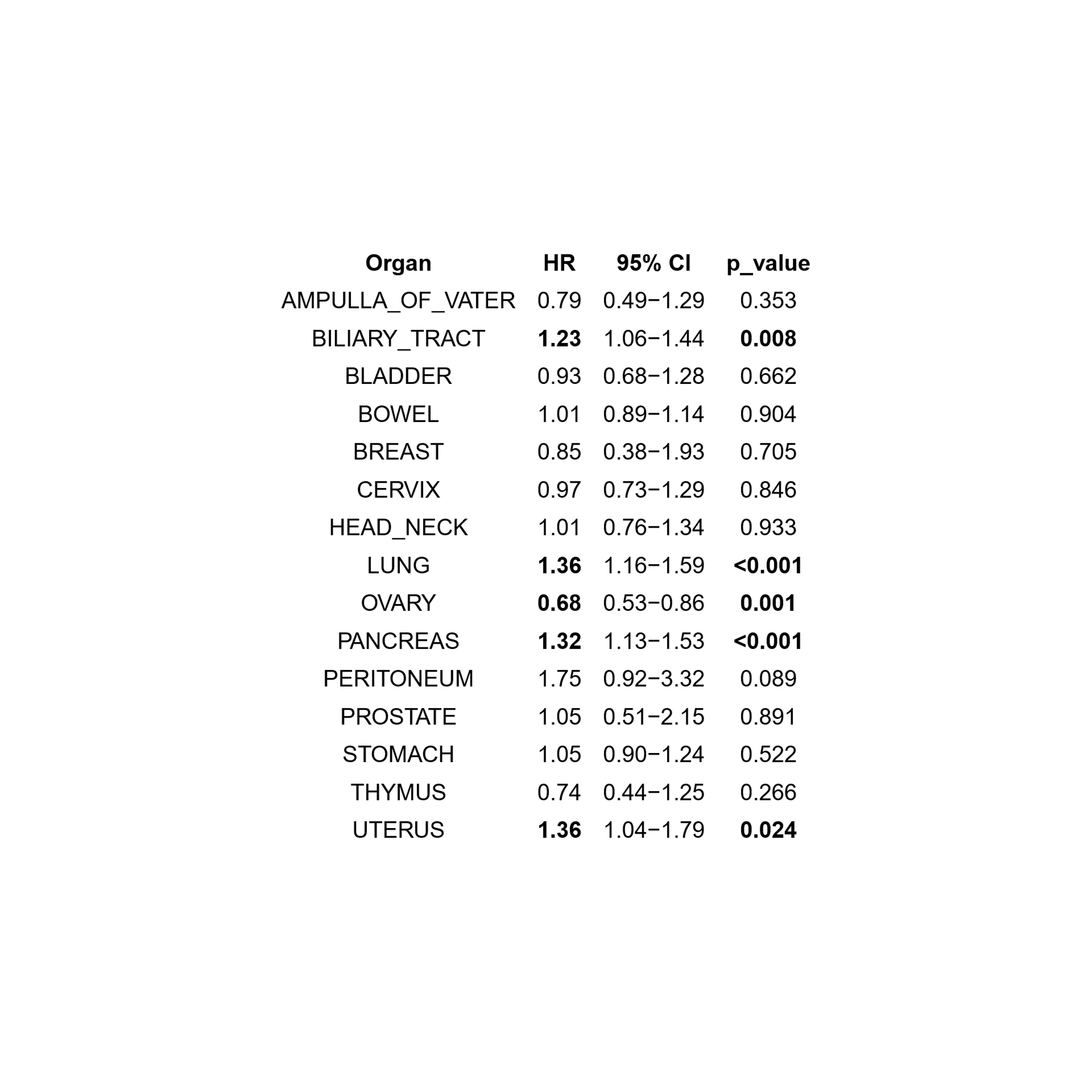


(B)

**
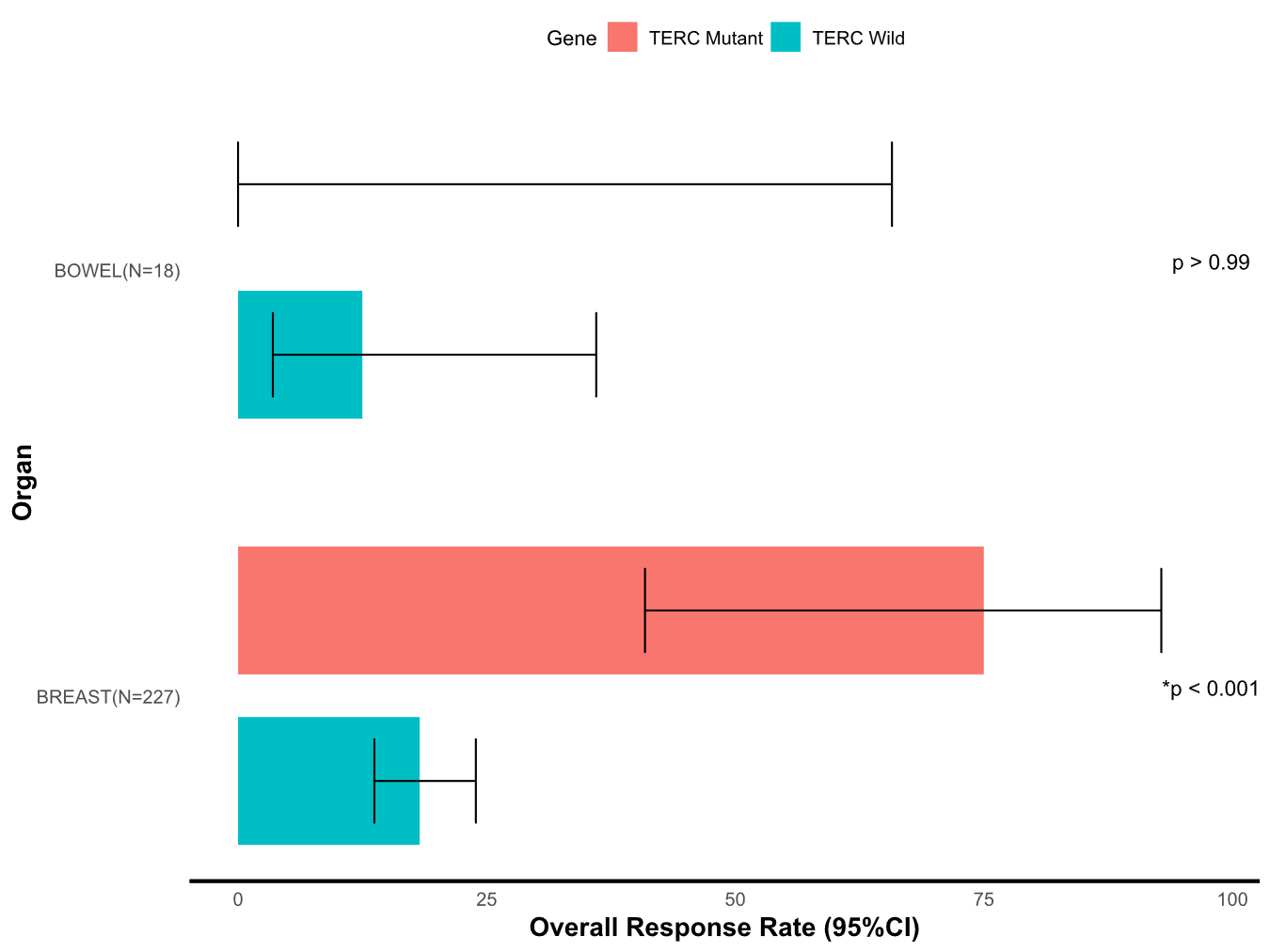
**


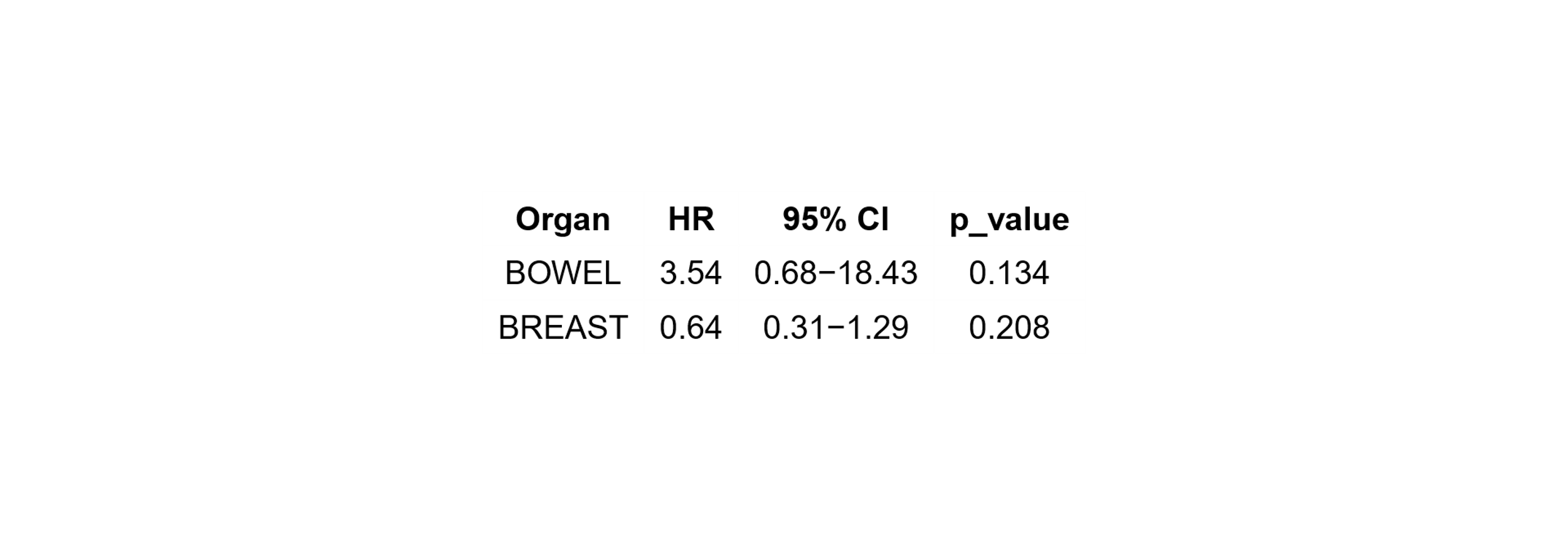


(C)


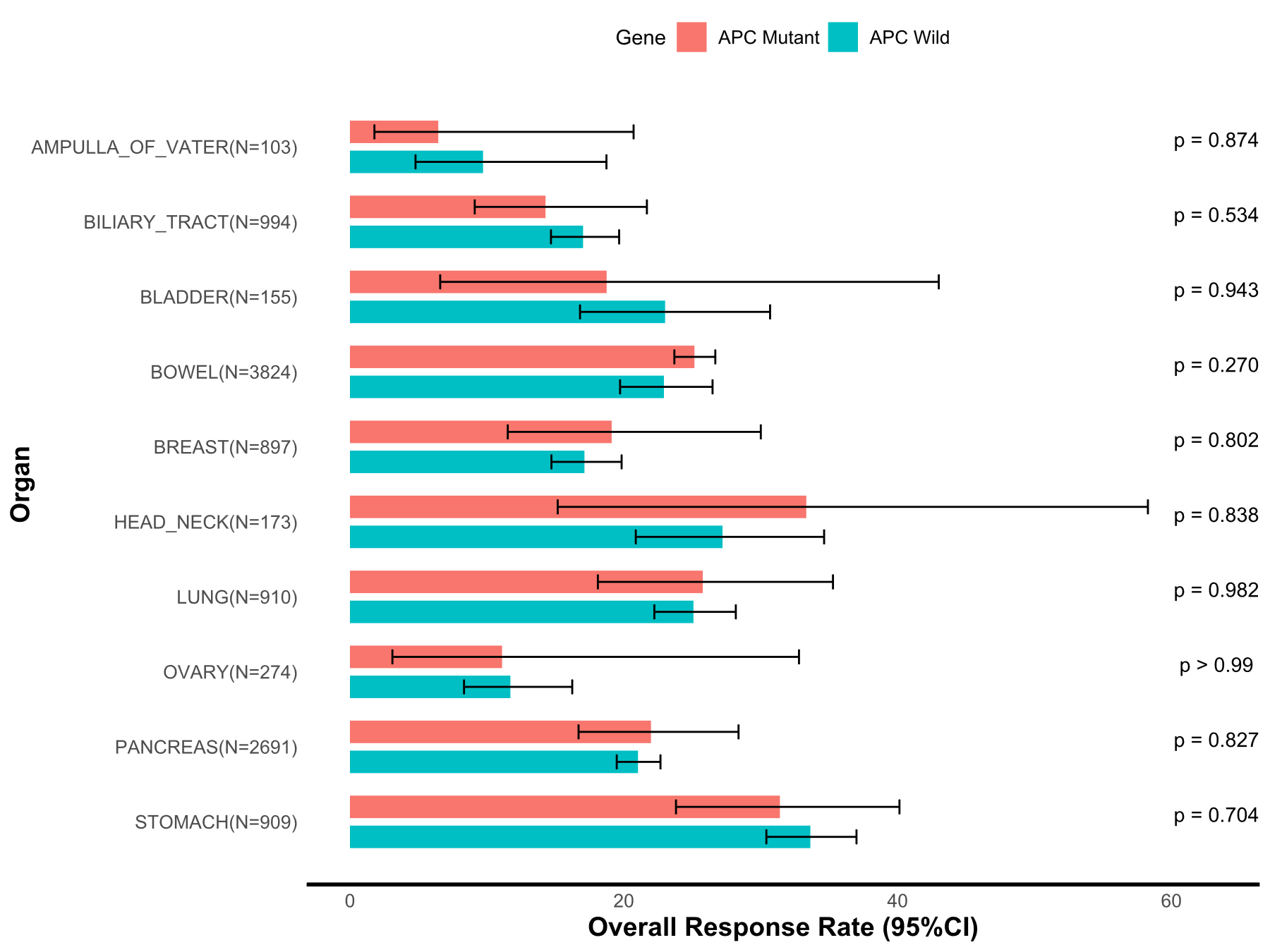


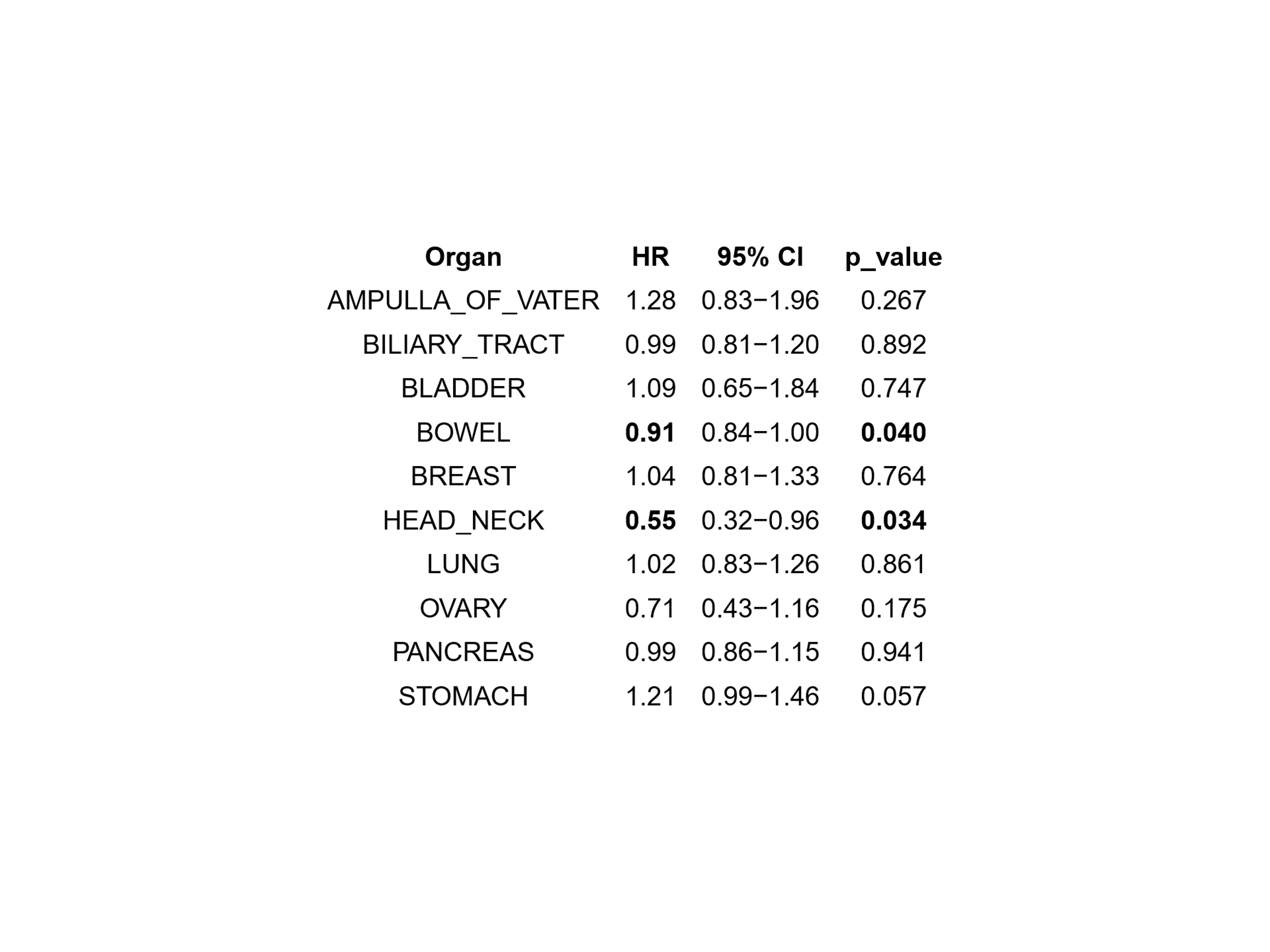


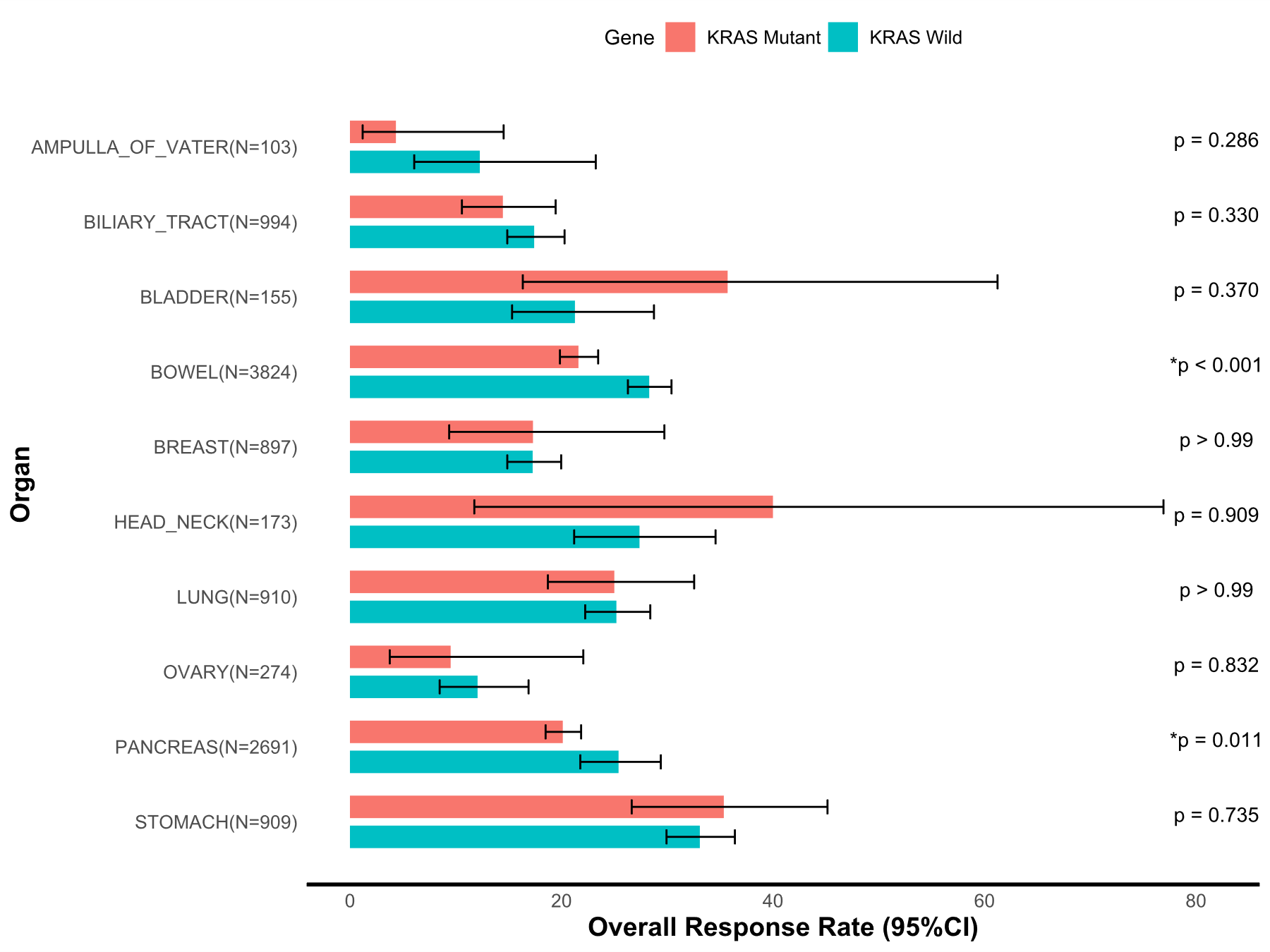


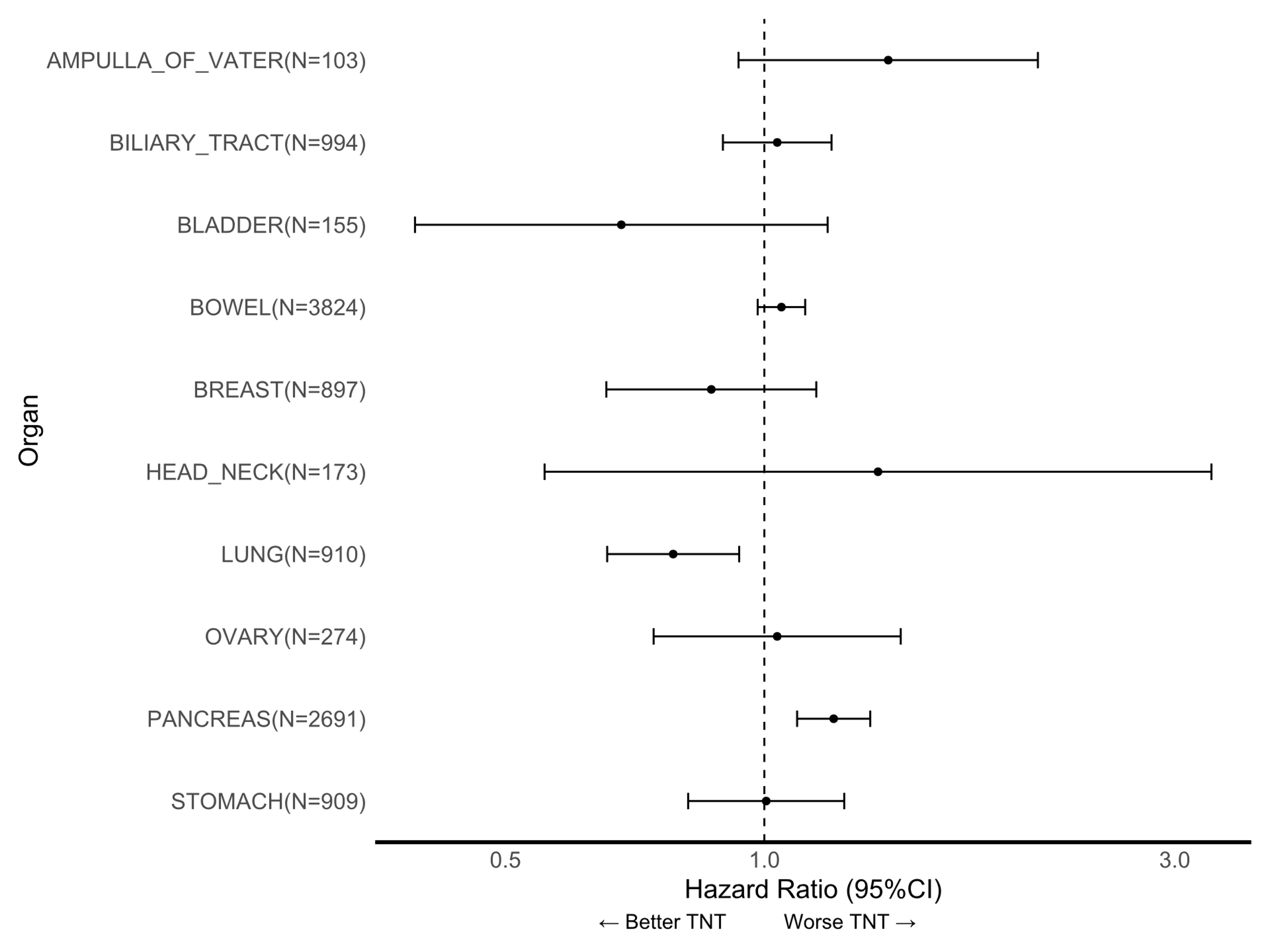

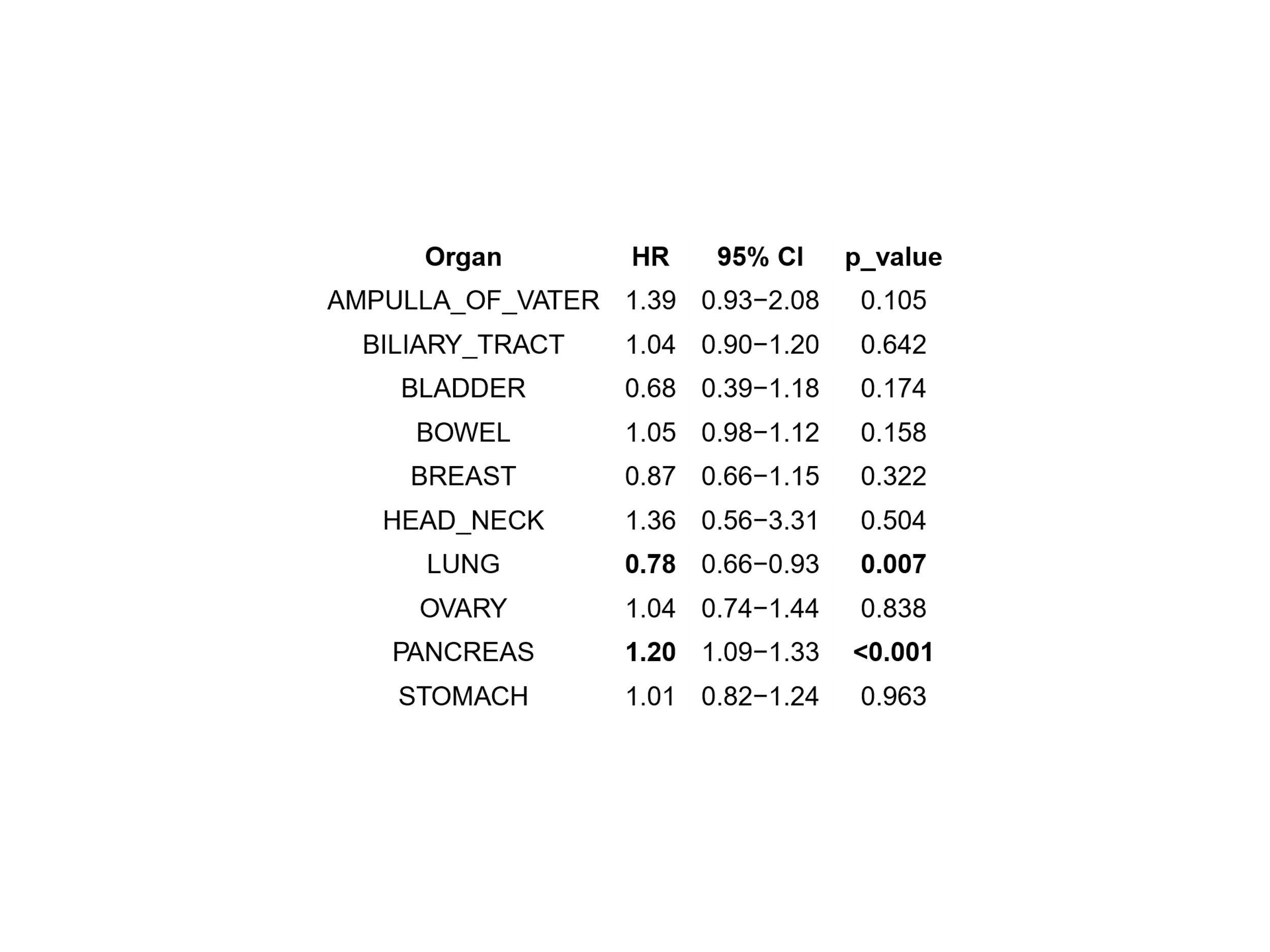


(D)

**
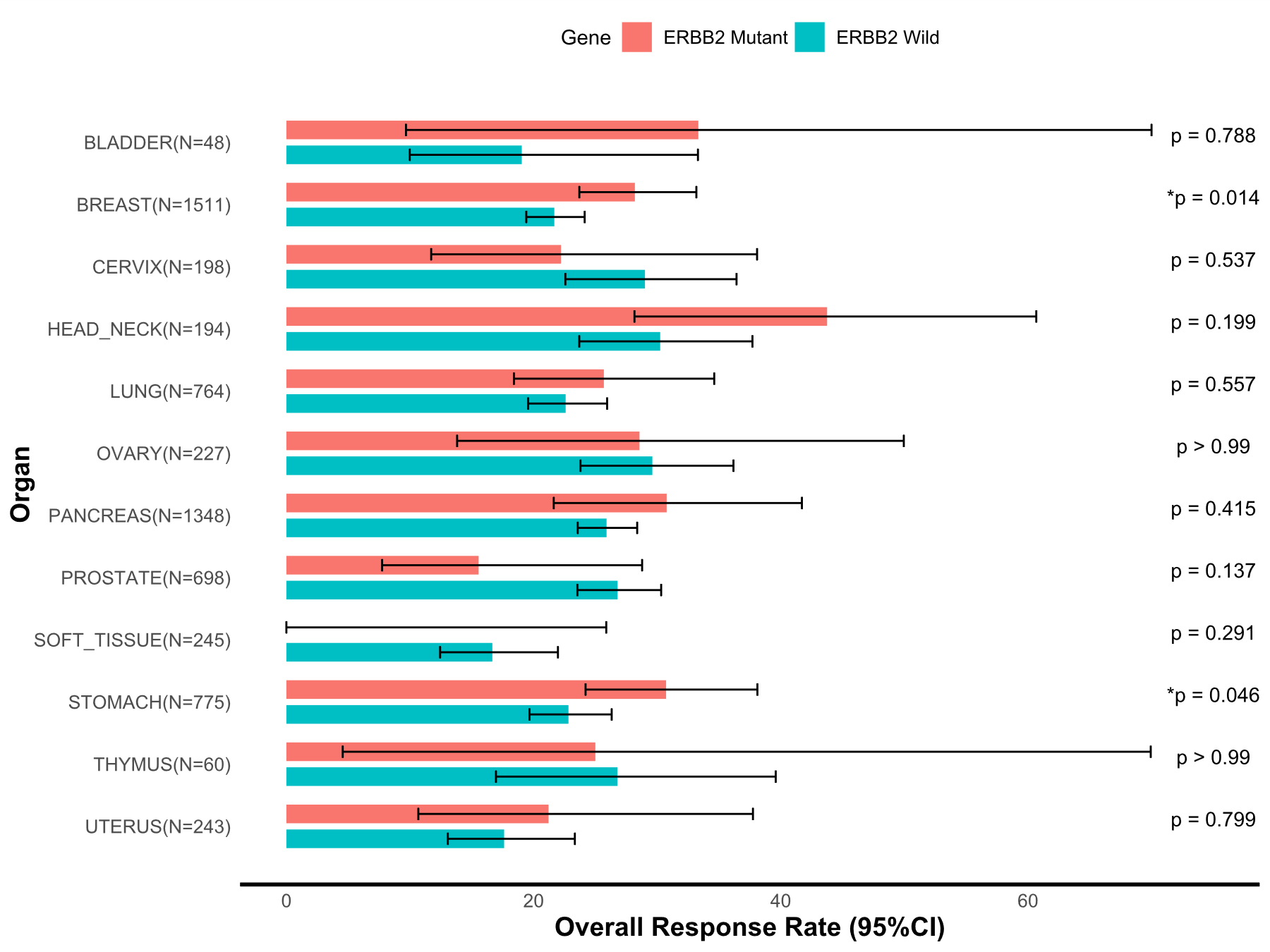
**


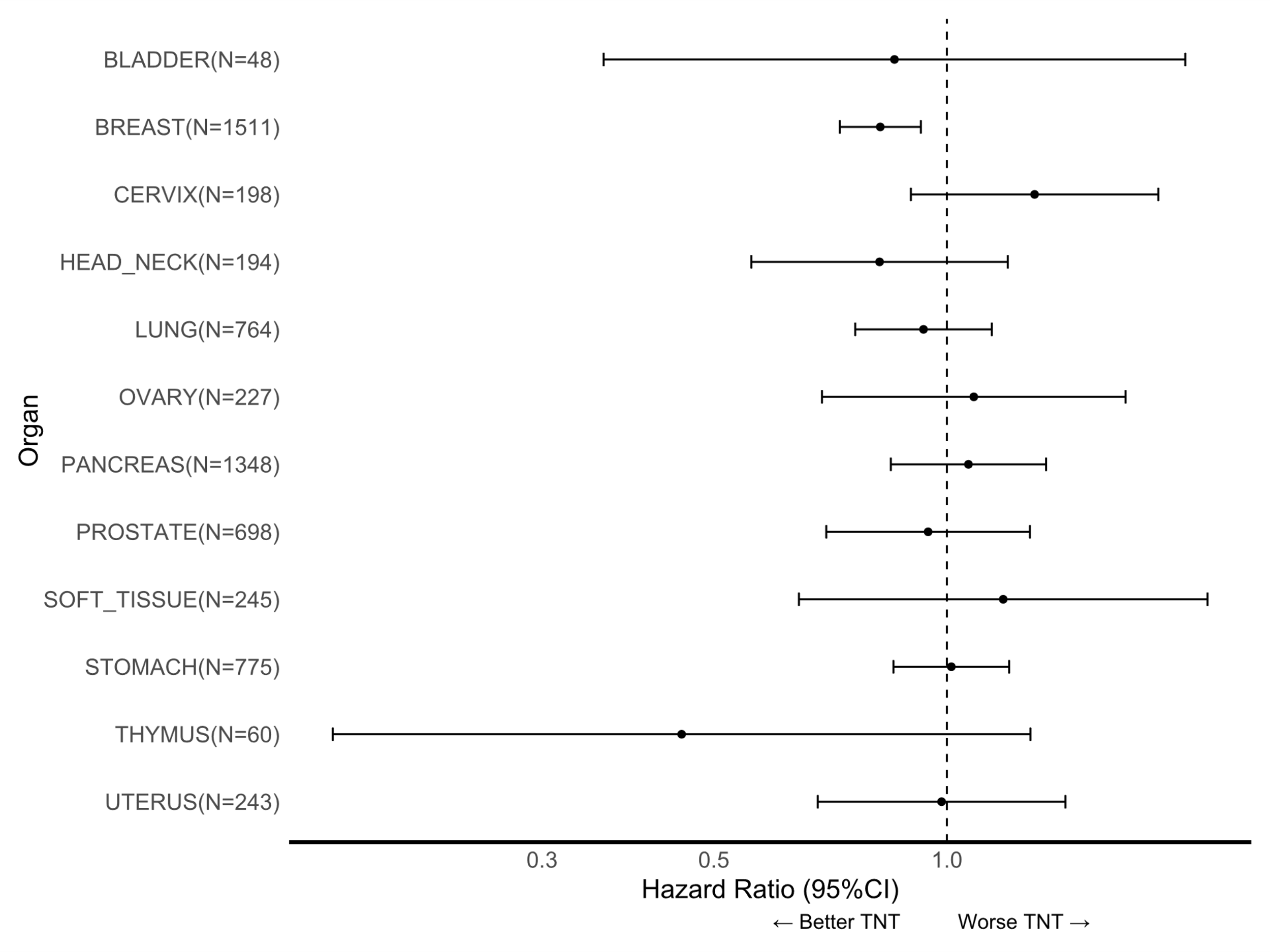

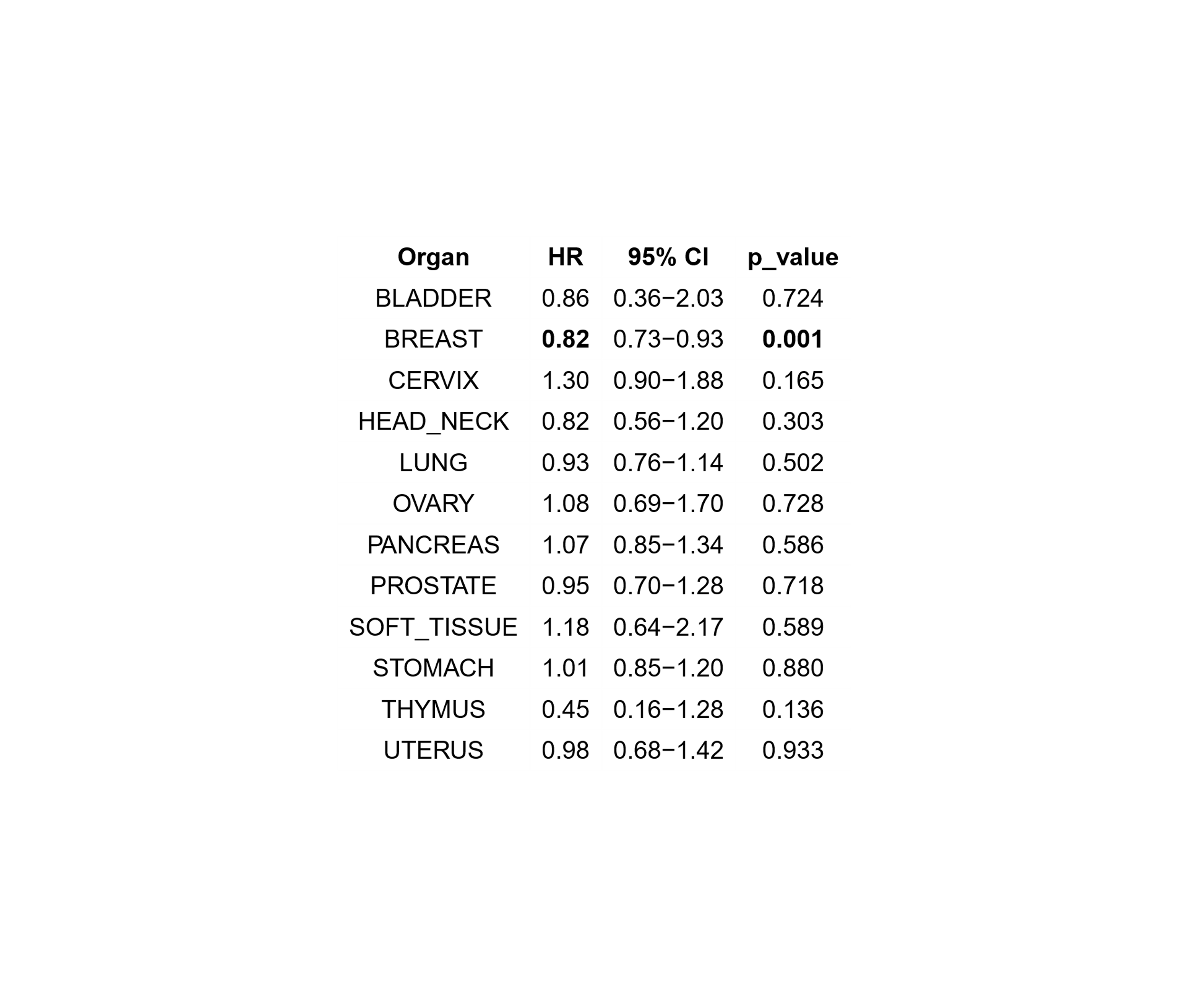


**
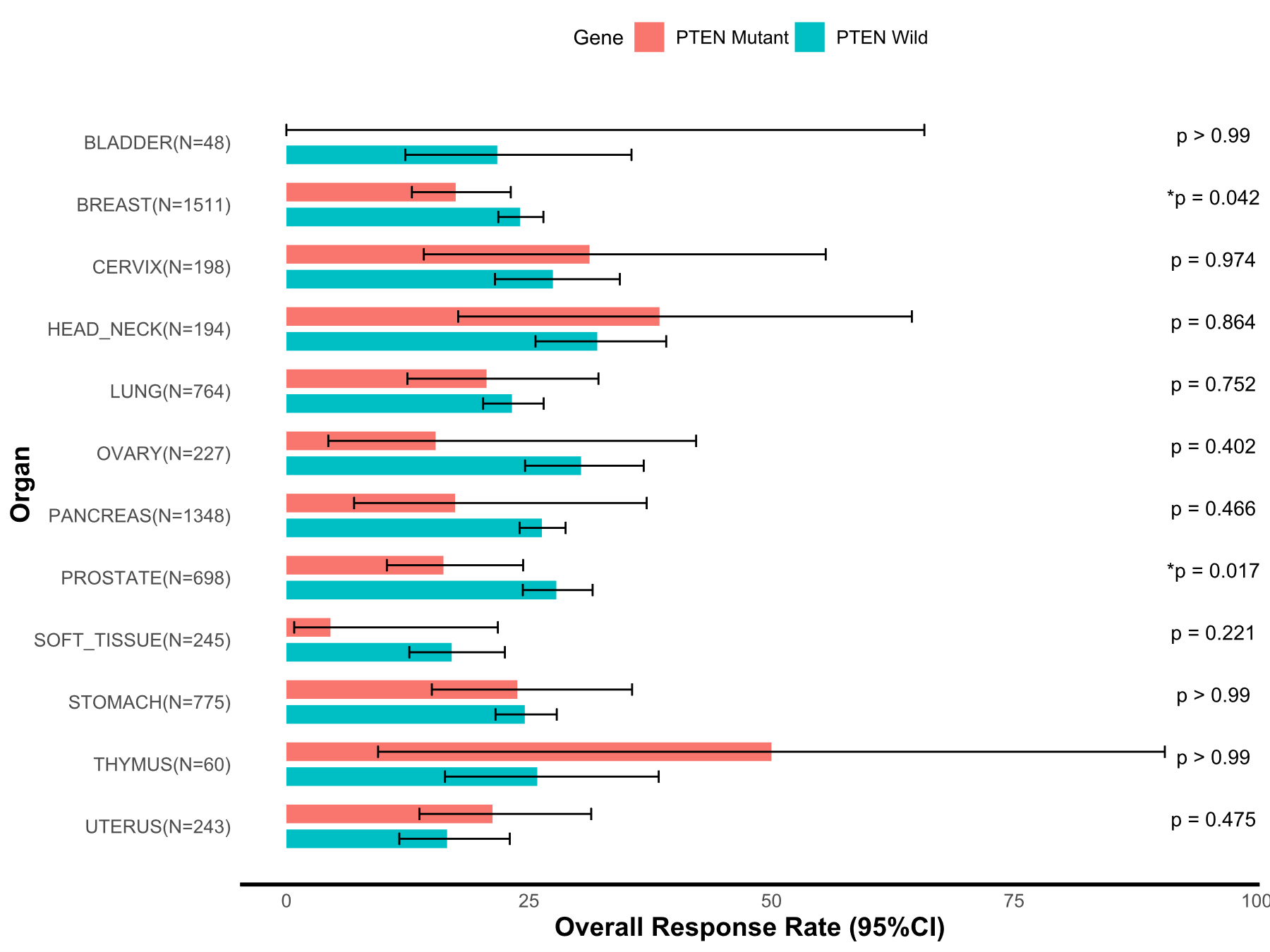
**


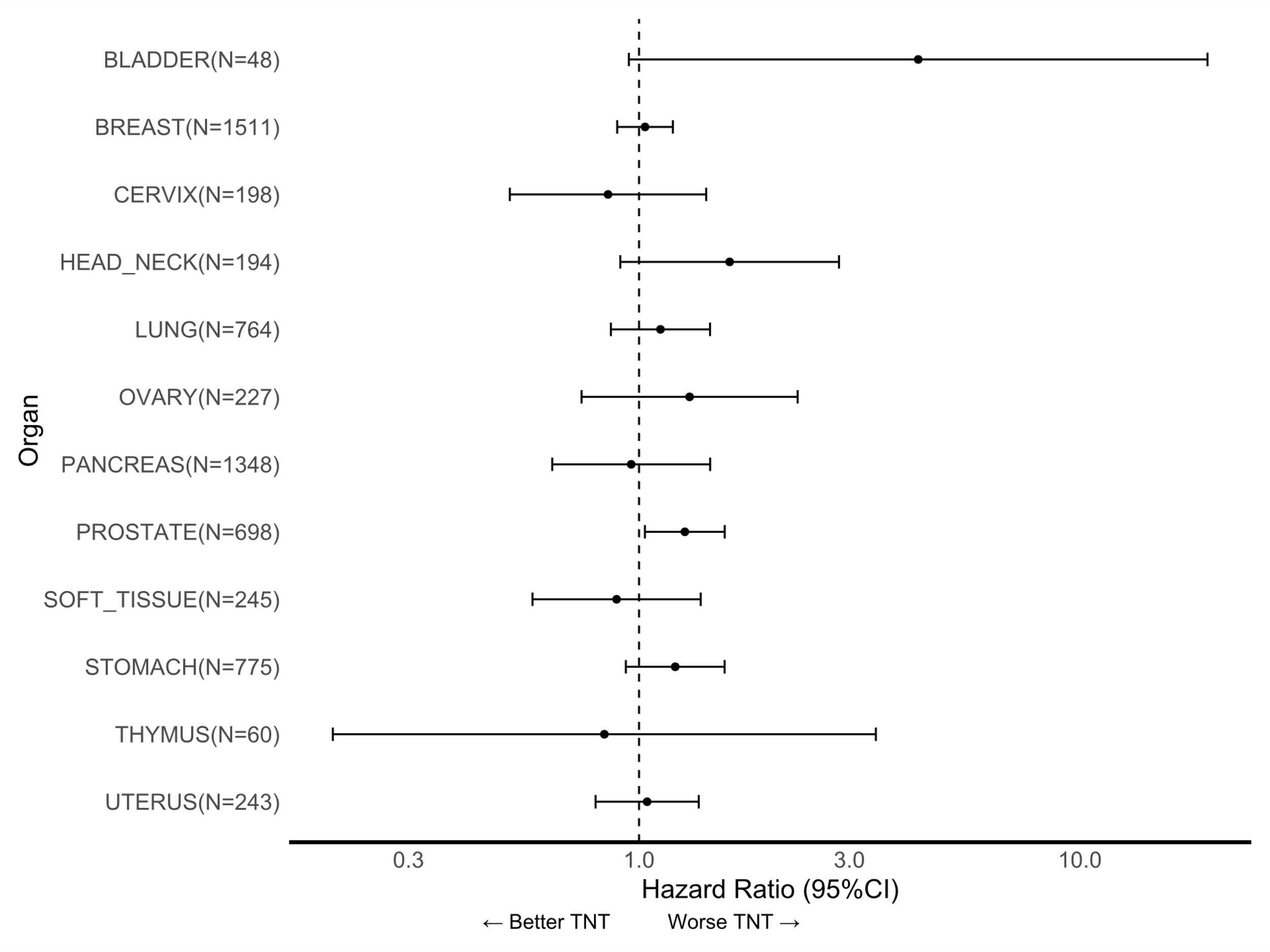

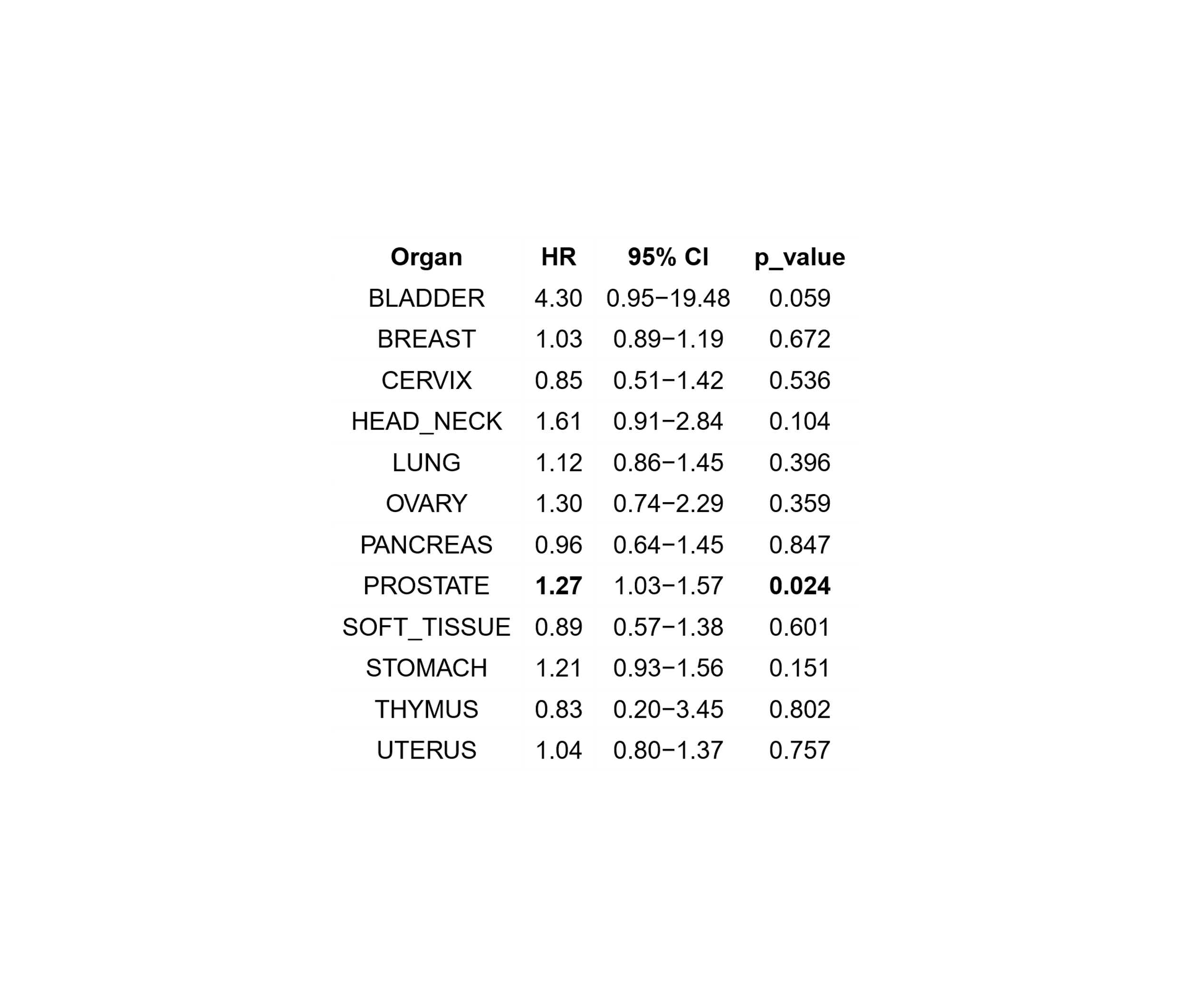


(E)

**
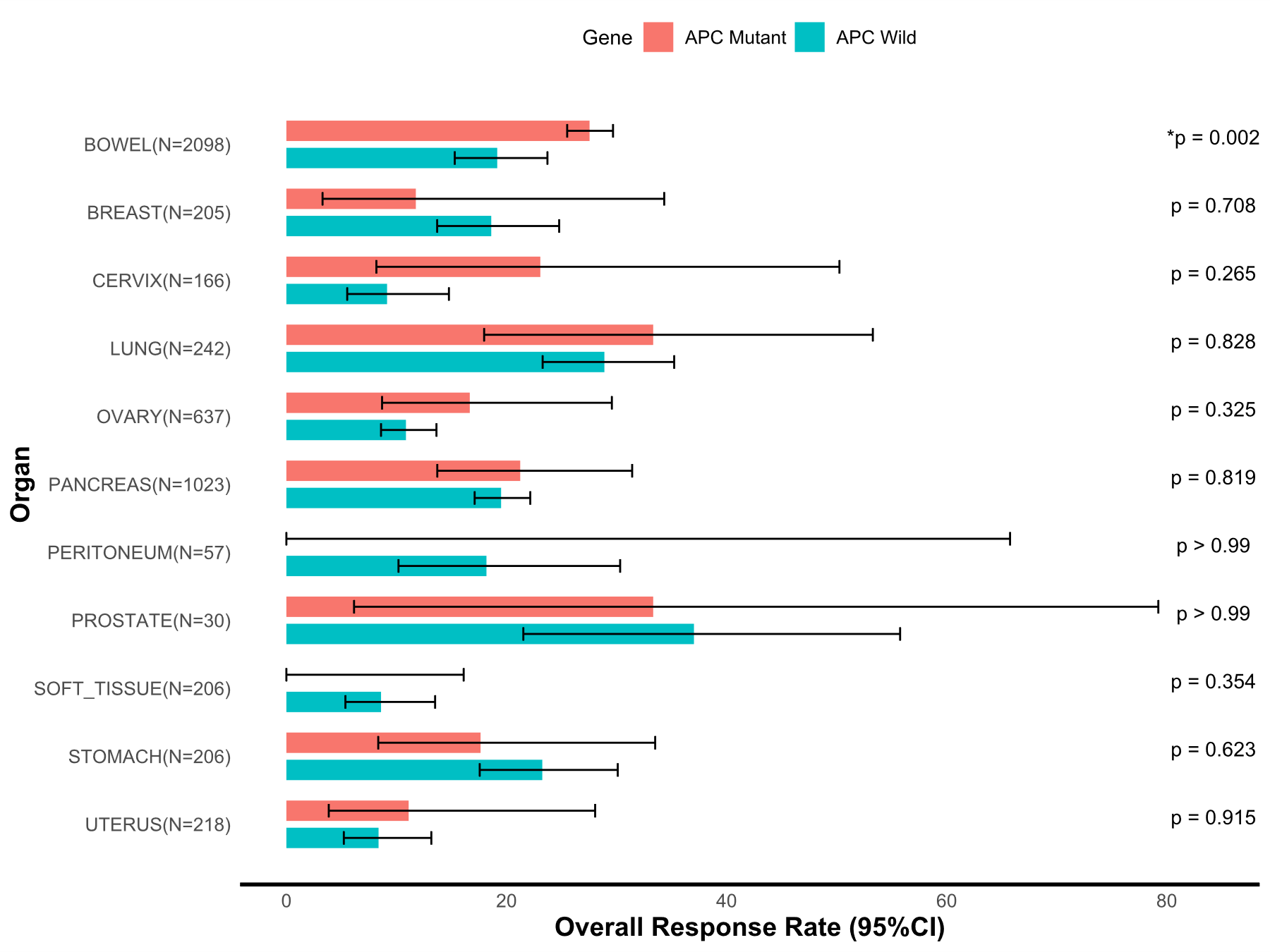
**


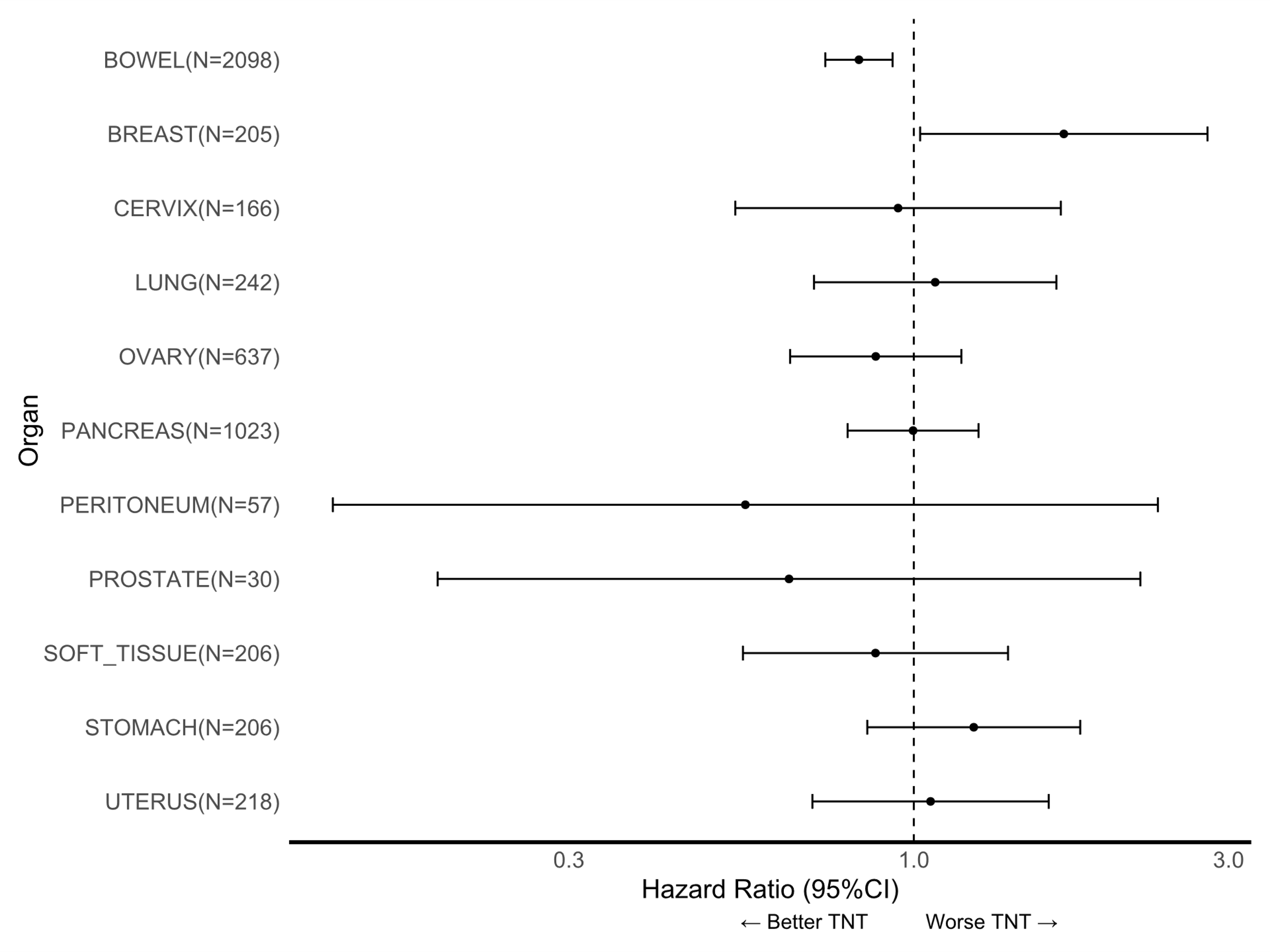

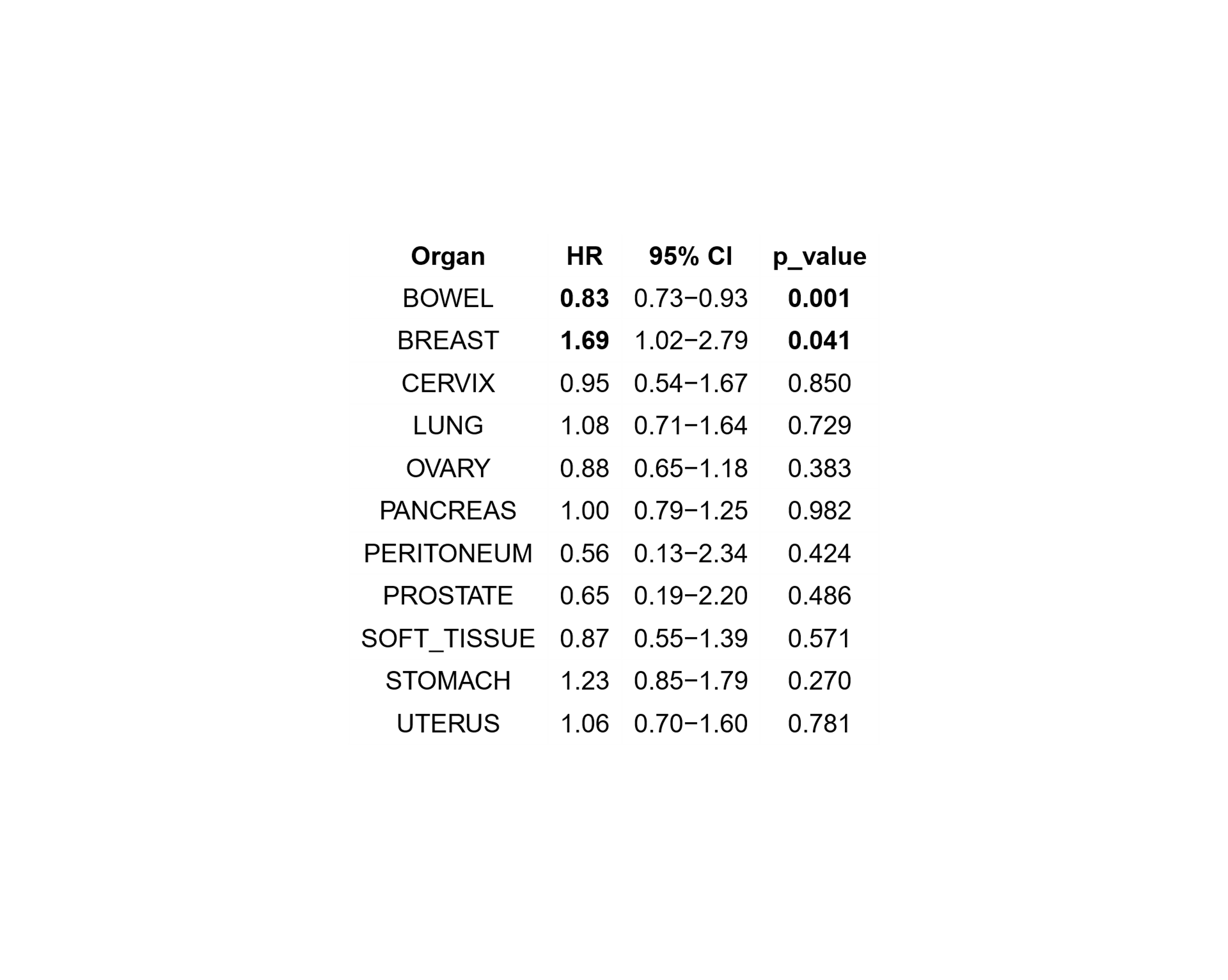


**
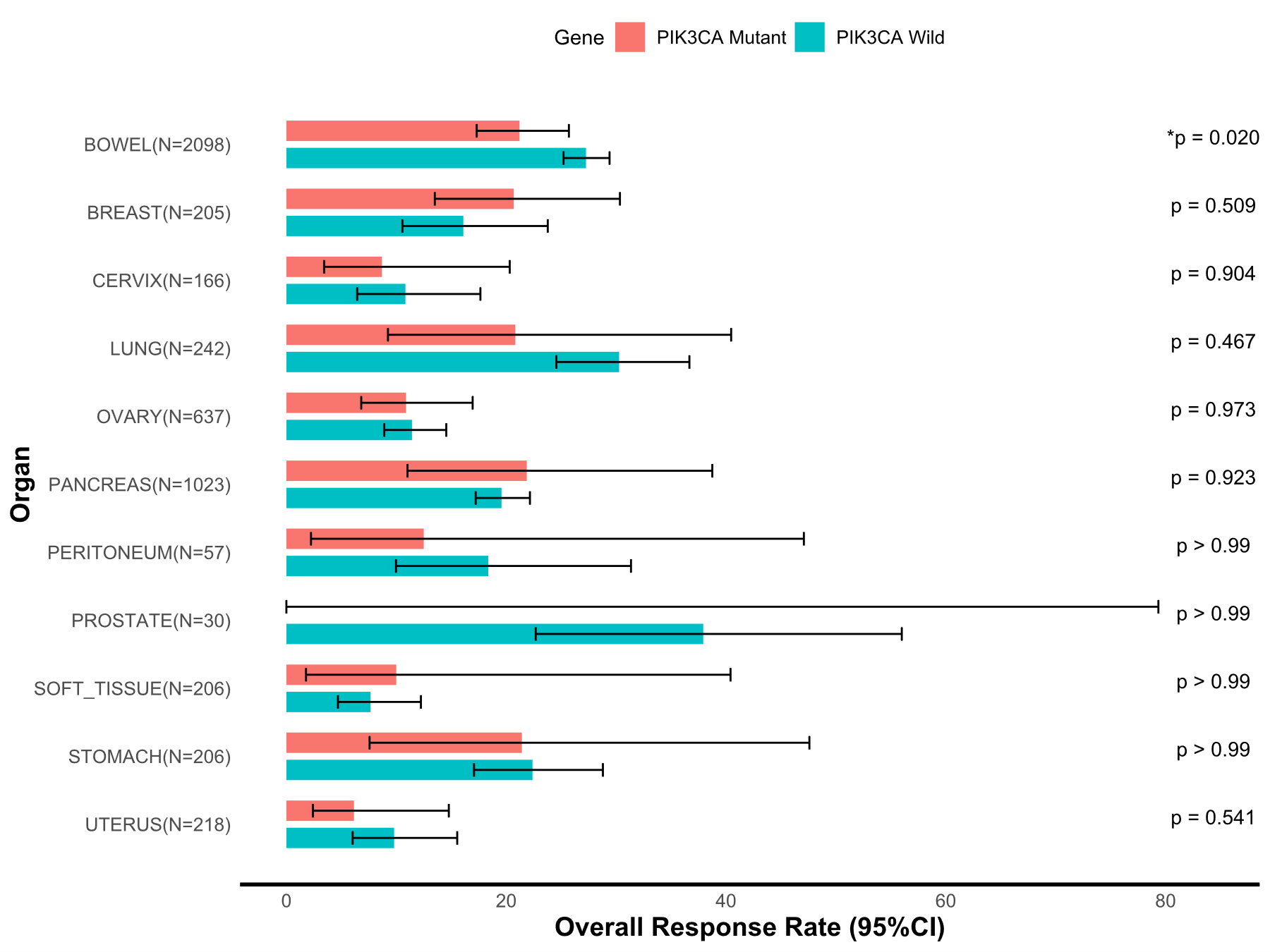
**


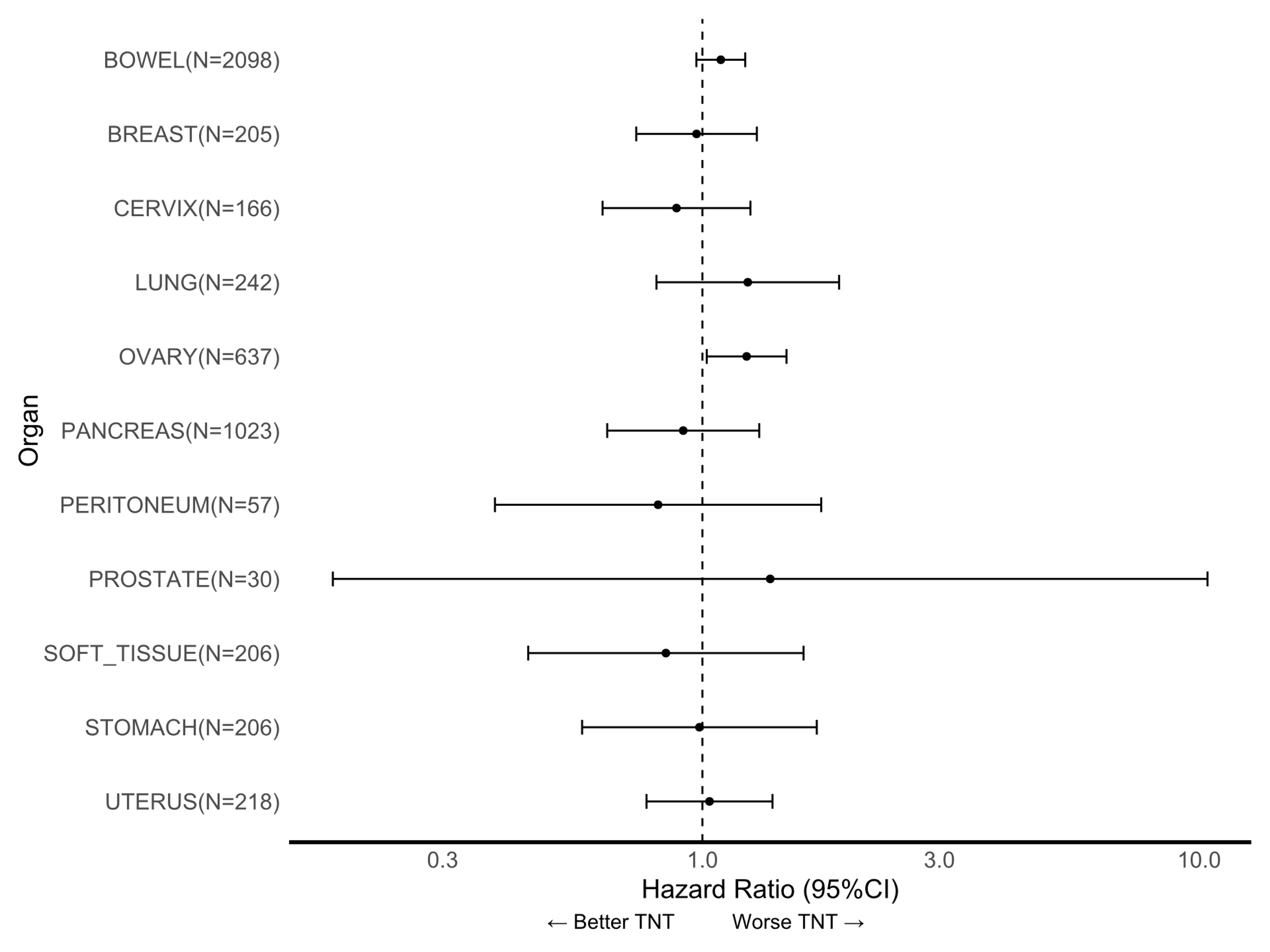

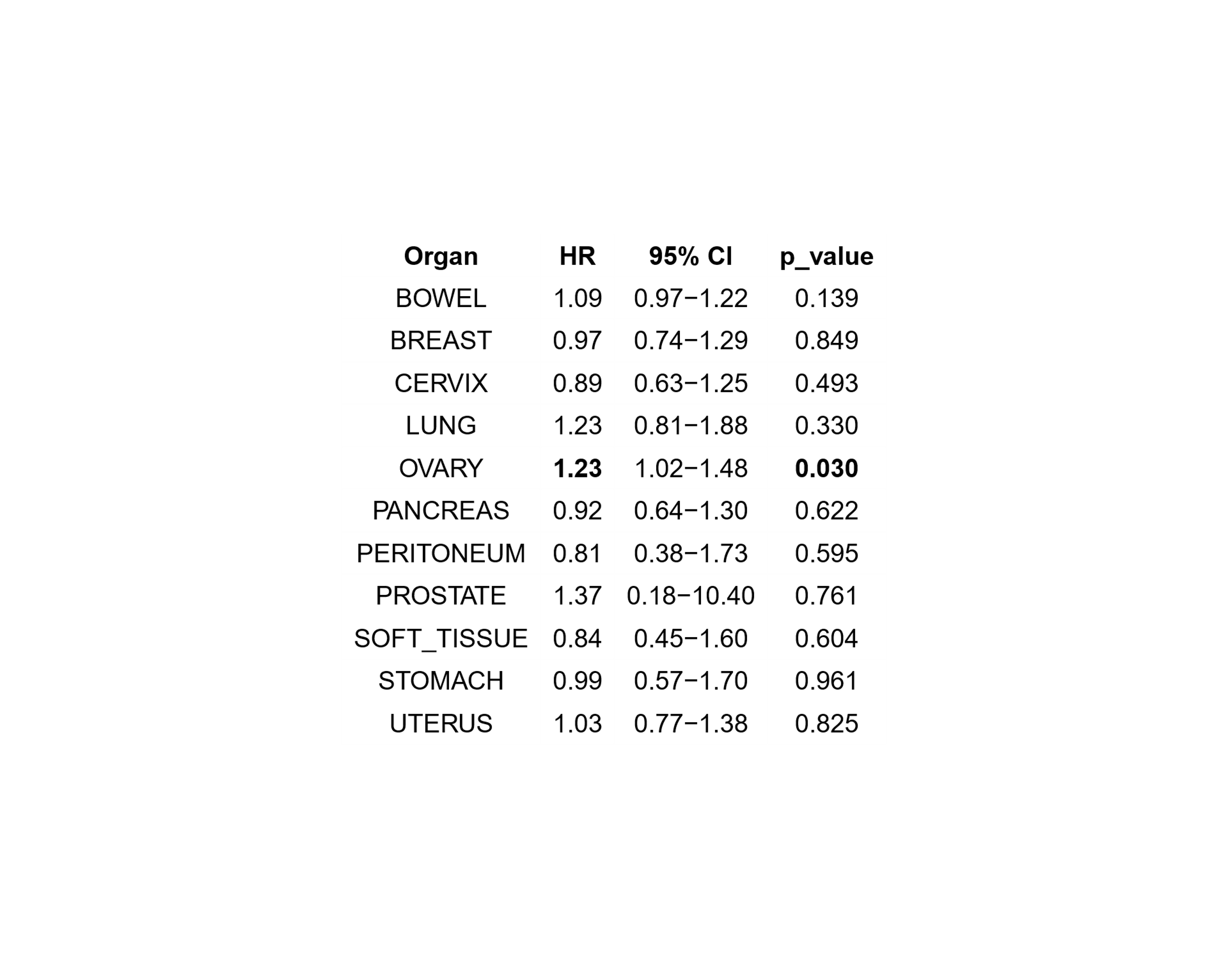


**
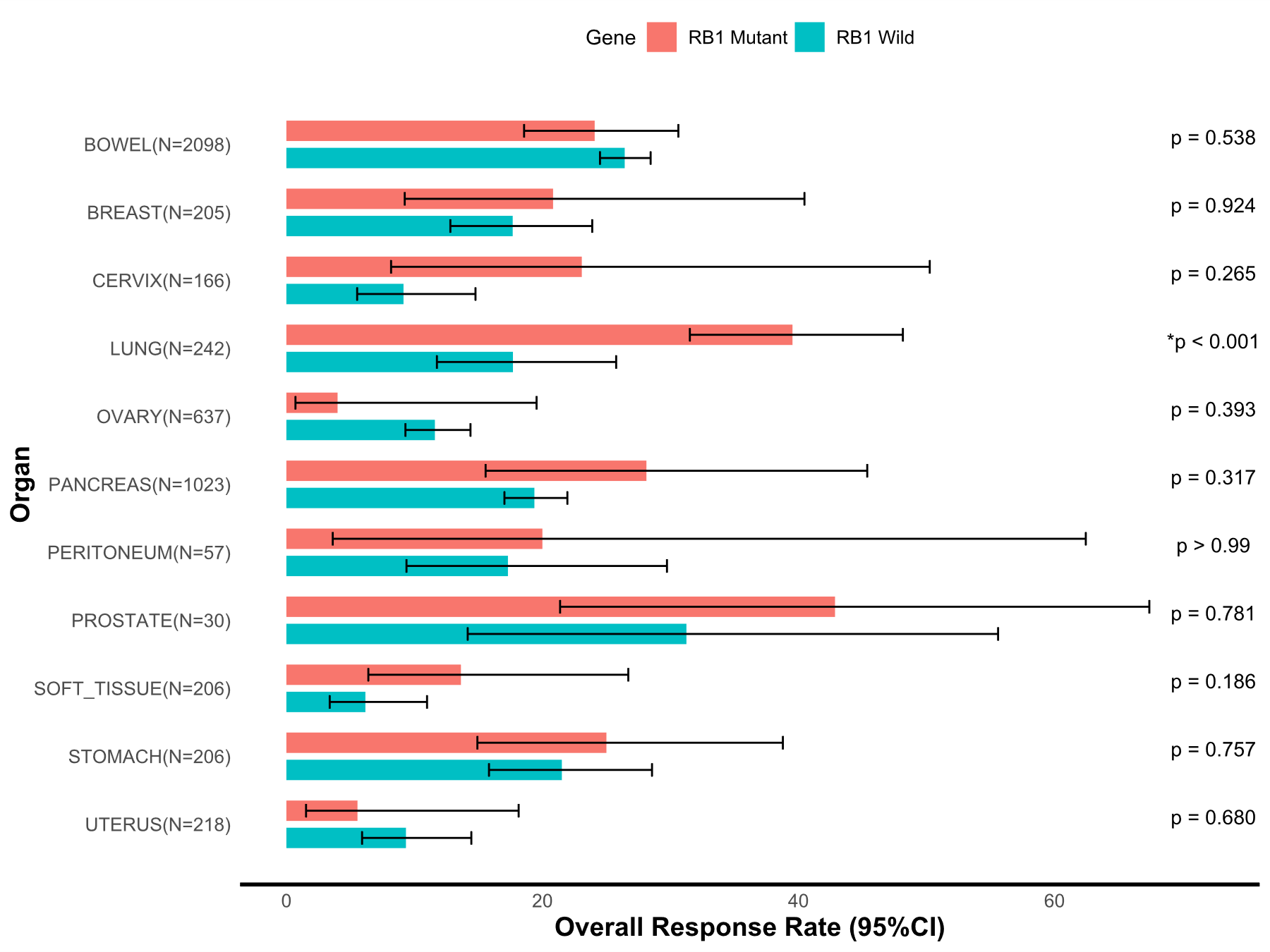
**


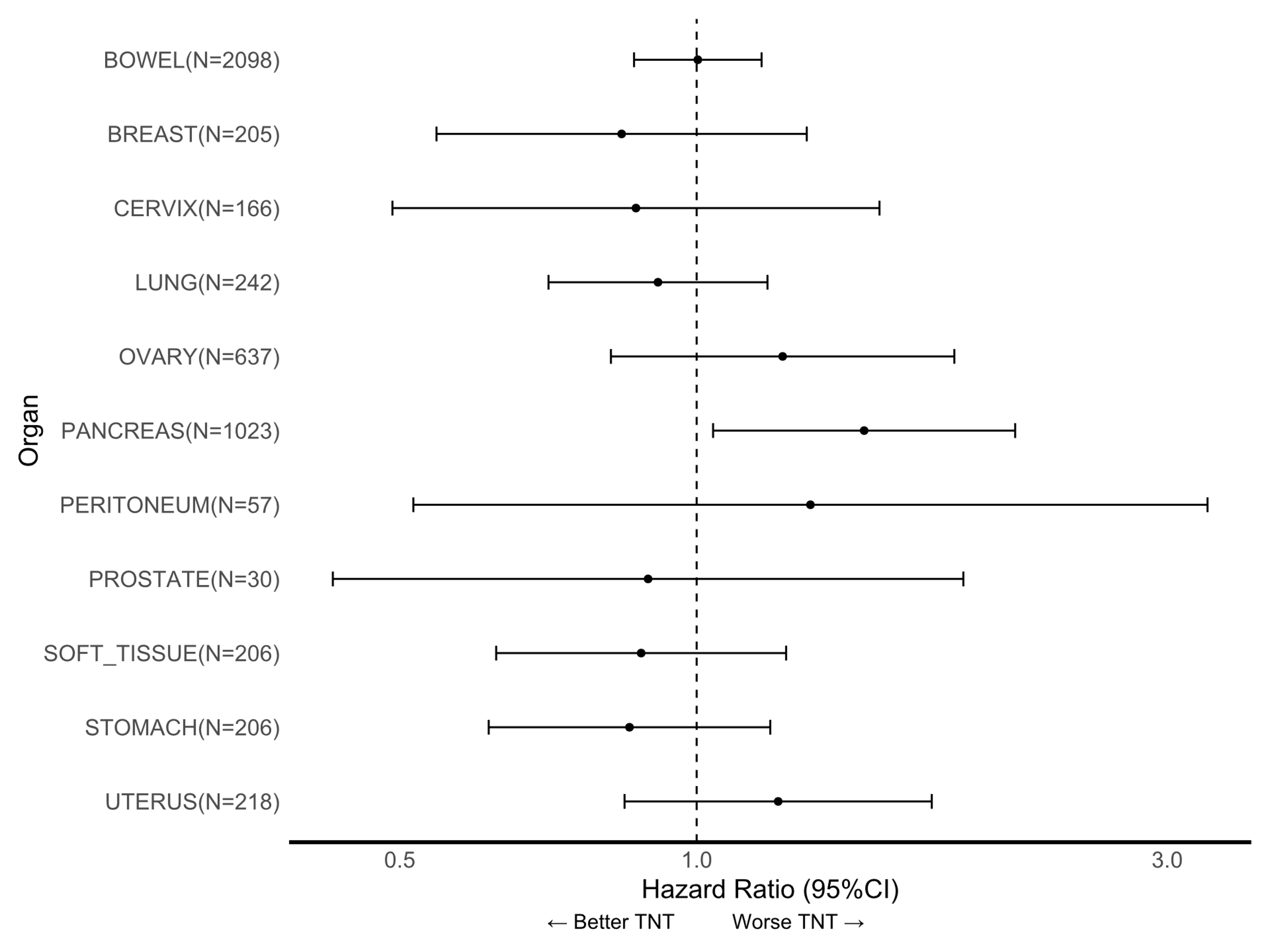

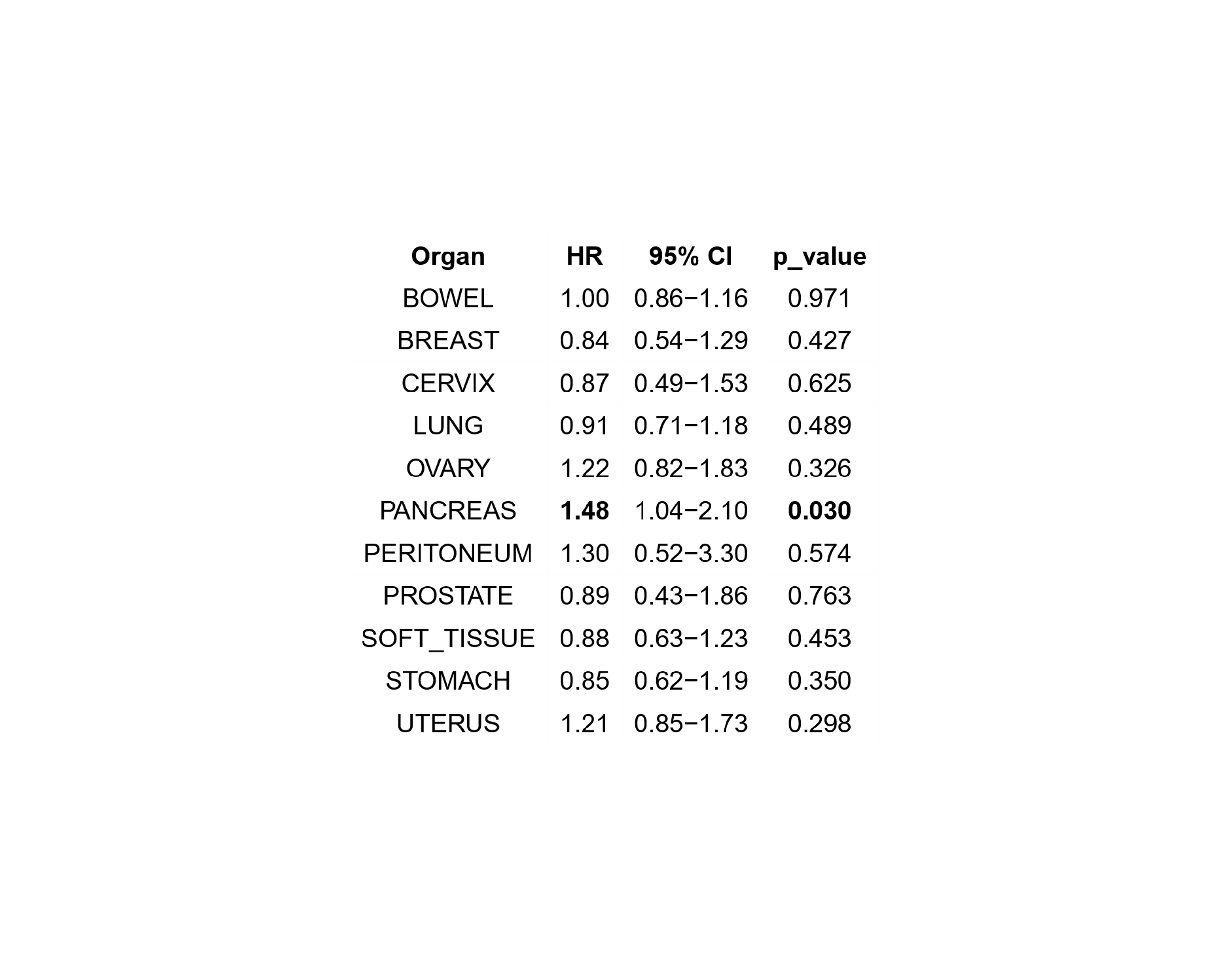


**
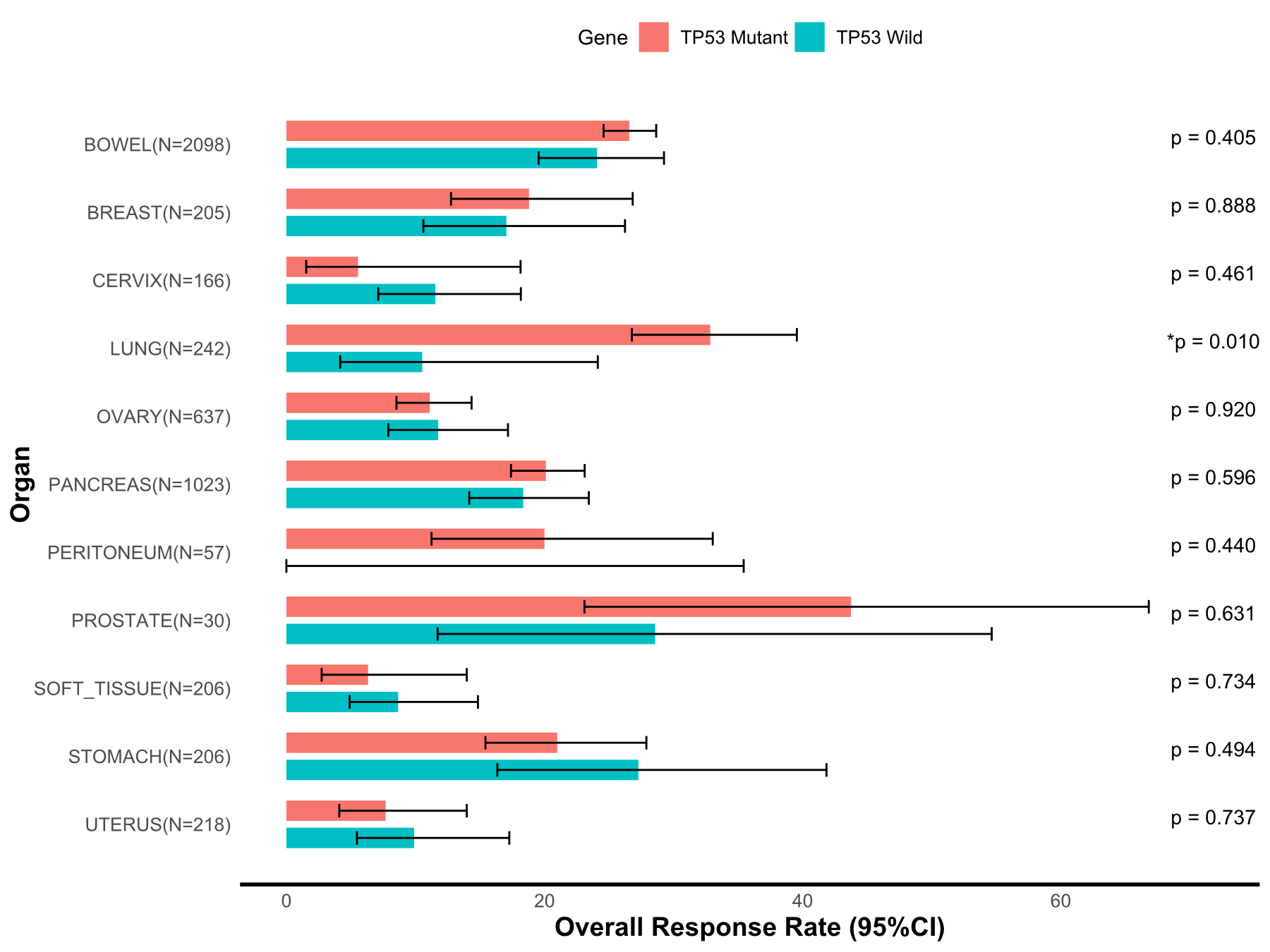
**


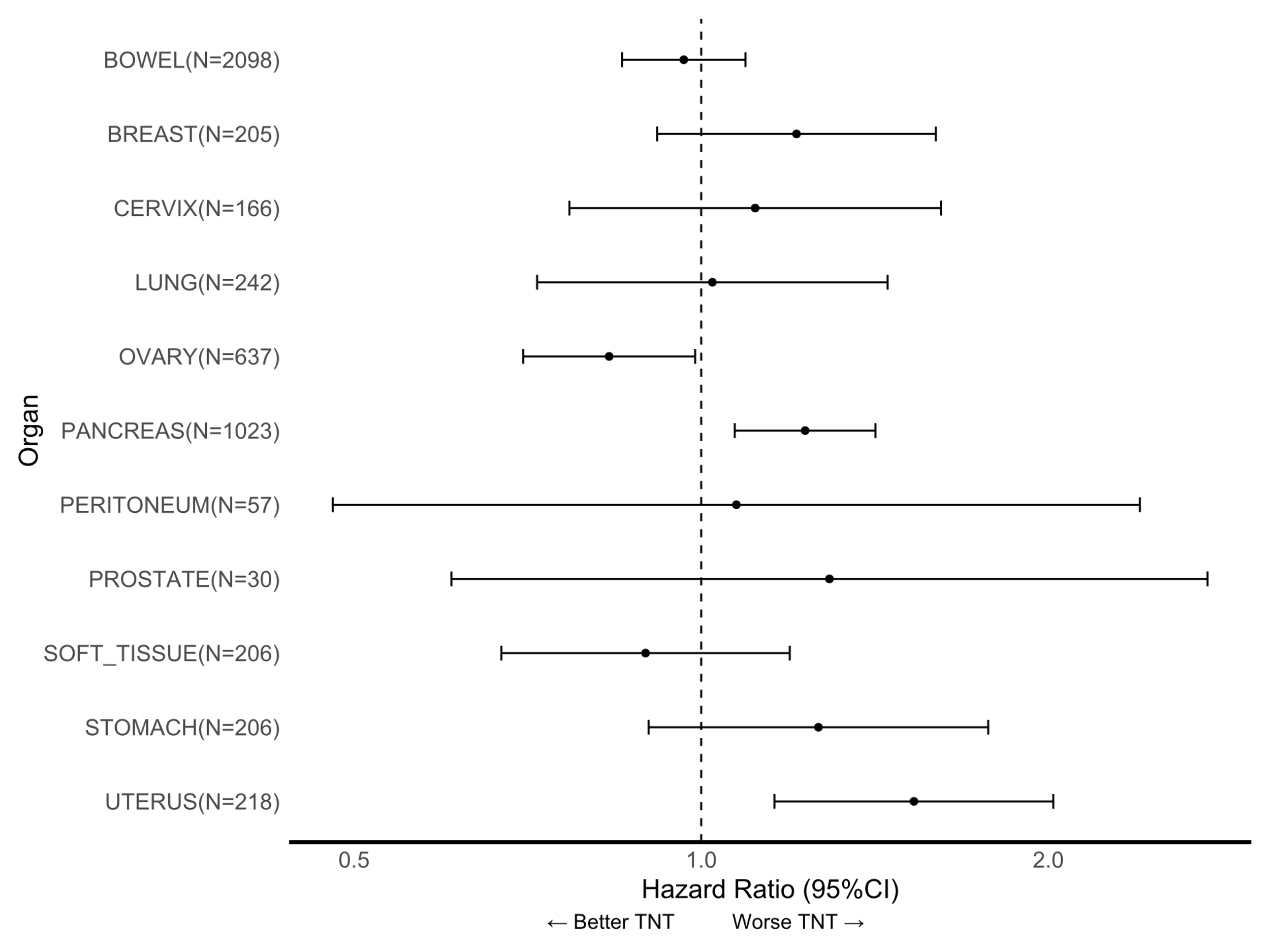

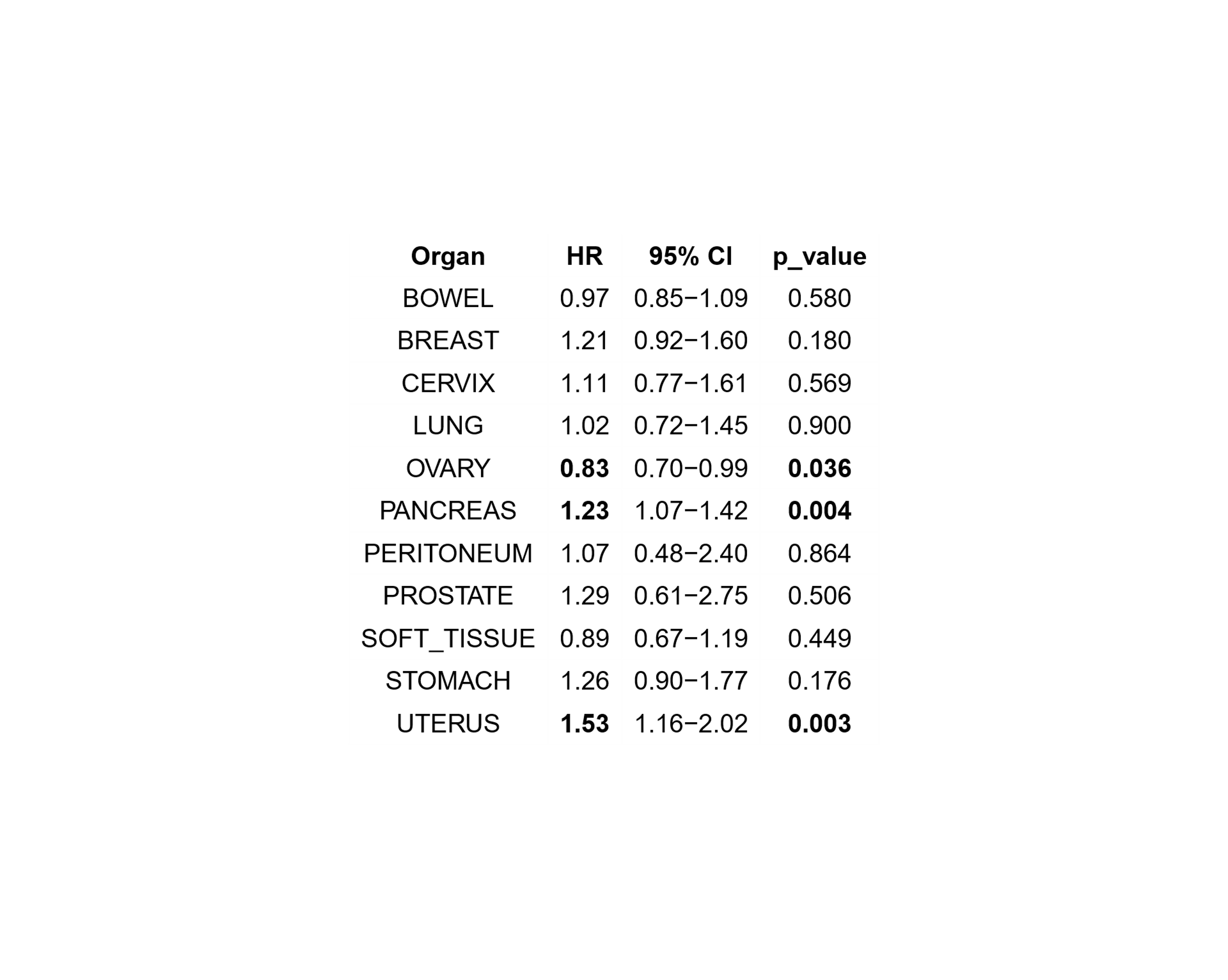


**Supplementary Figure3: Subgroup Analysis of Receiver Operating Characteristic (ROC) curves of overall response rate for each prediction model. ROC curves illustrate the predictive performance of different models across five drug categories: A, Platinum-based drugs; B, Alkylating agents; C, Antimetabolites; D, Microtubule inhibitors; E, Topoisomerase inhibitors. Individual ROC curves and the mean ROC curve for each model are shown.**

**High-TMB(>10 mutations/Mb) group**

1. (B)


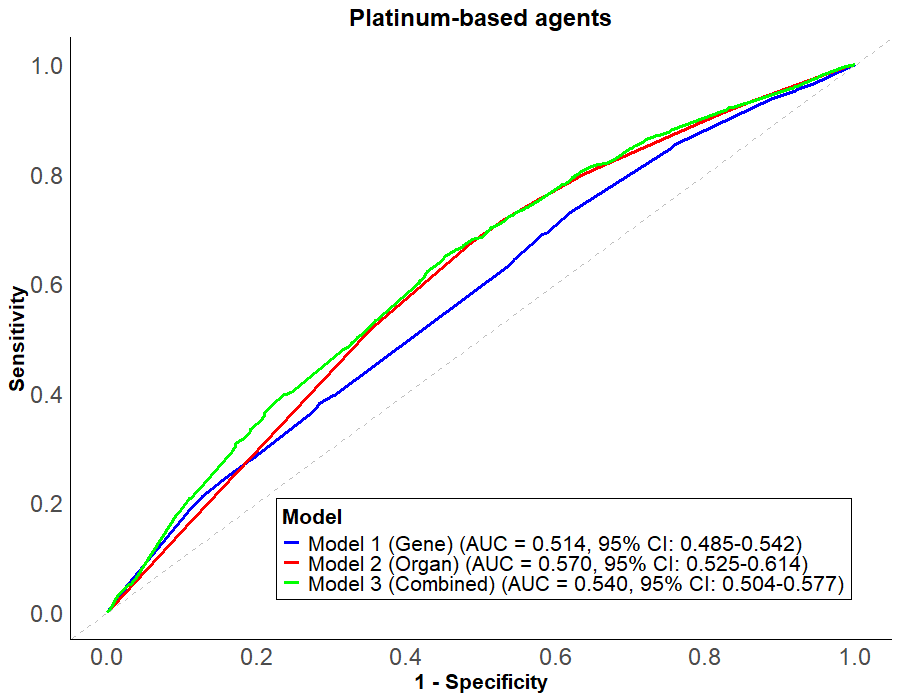

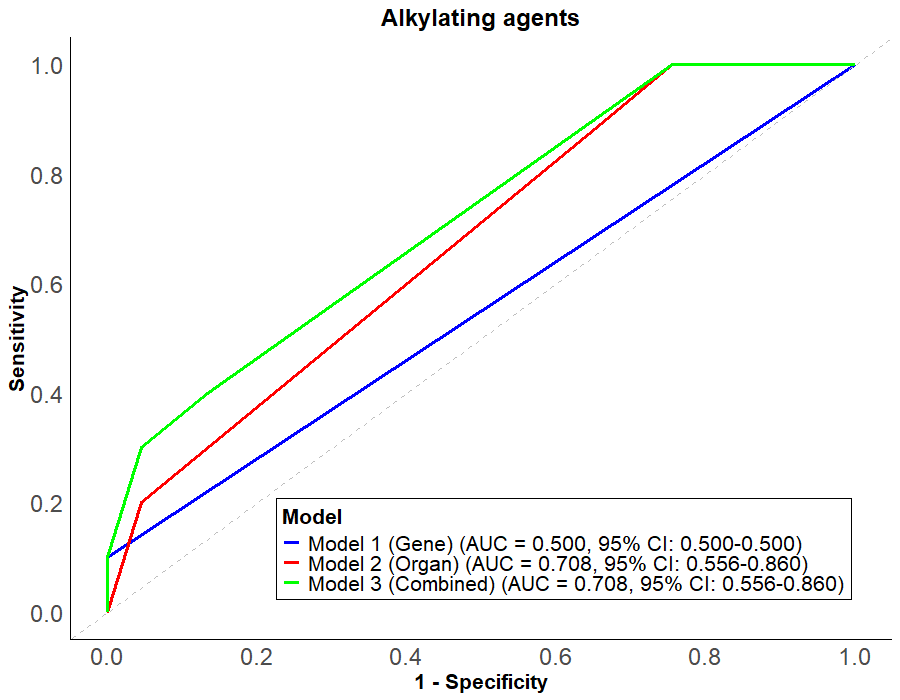


1. (D)


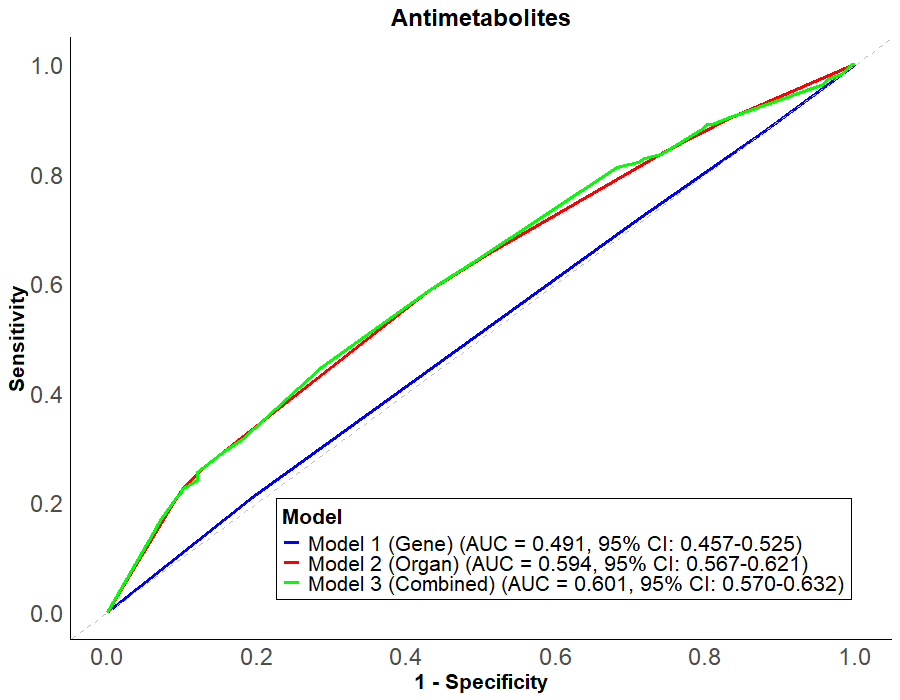

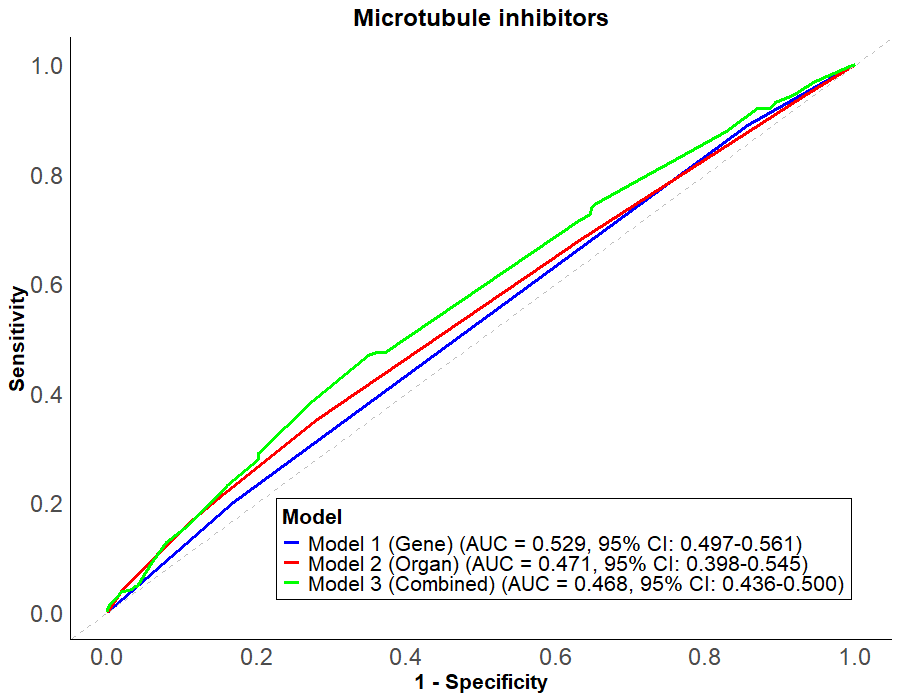


(E)


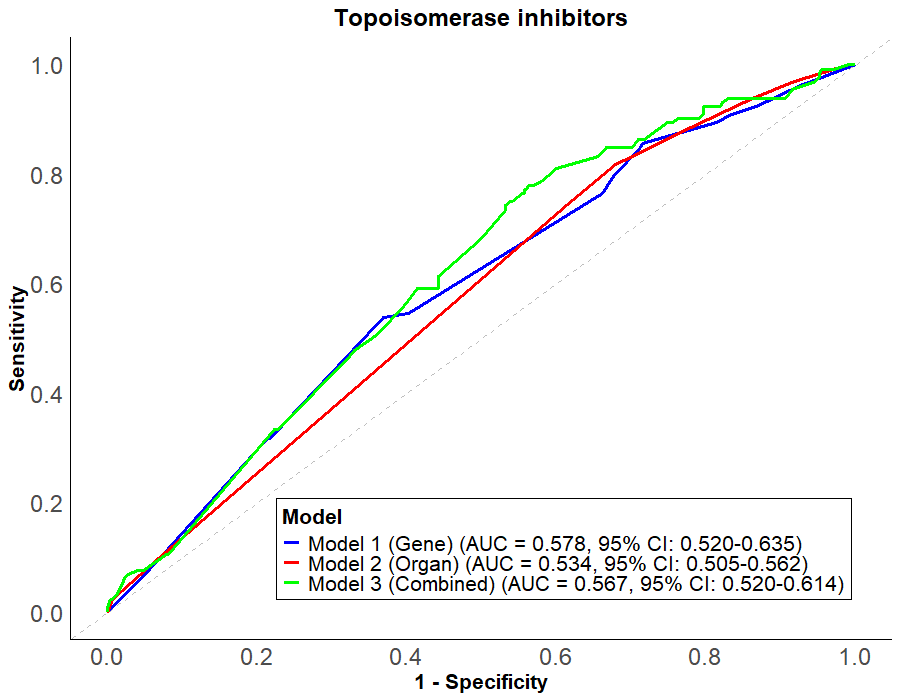


**Low-TMB(<5 mutations/Mb) group**

(A) (B)


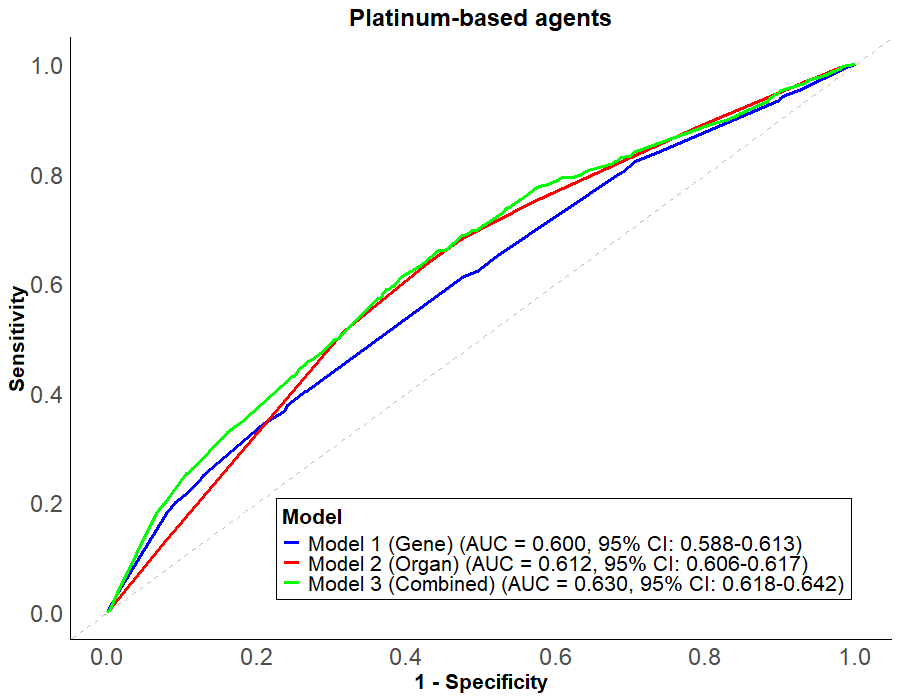

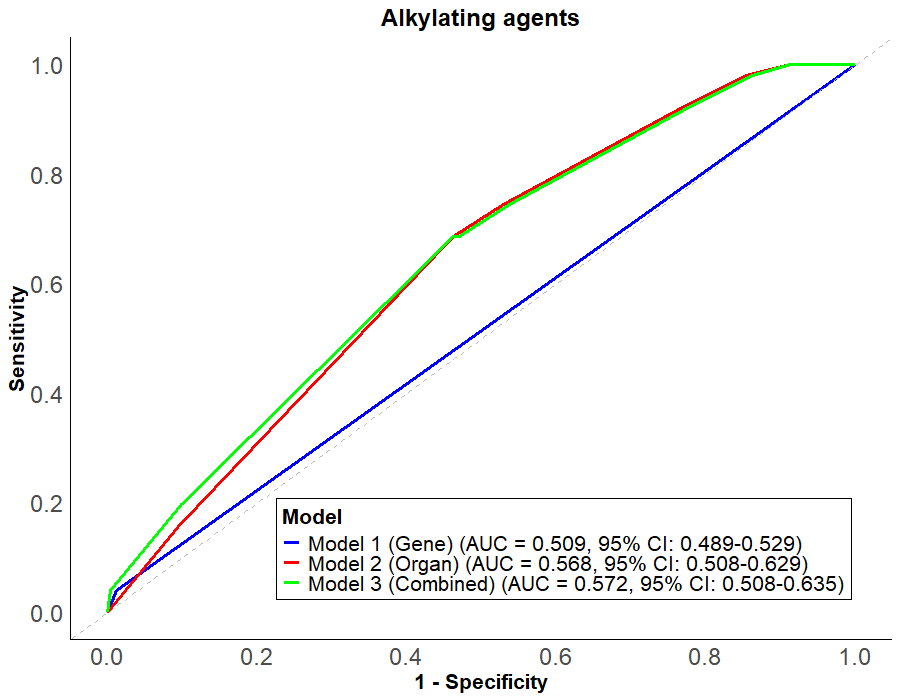


(C) (D)


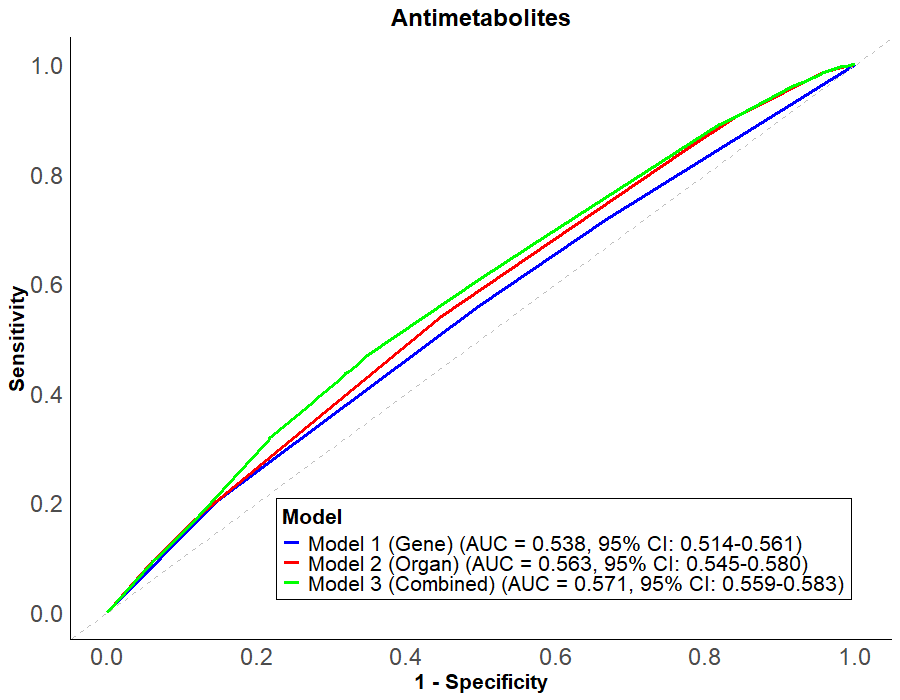

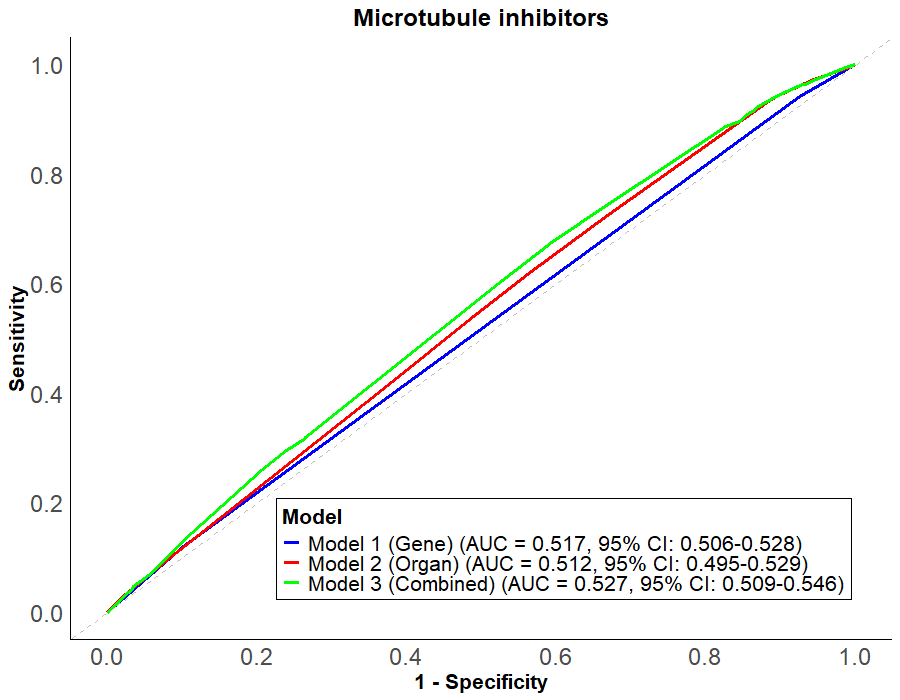


(E)

**
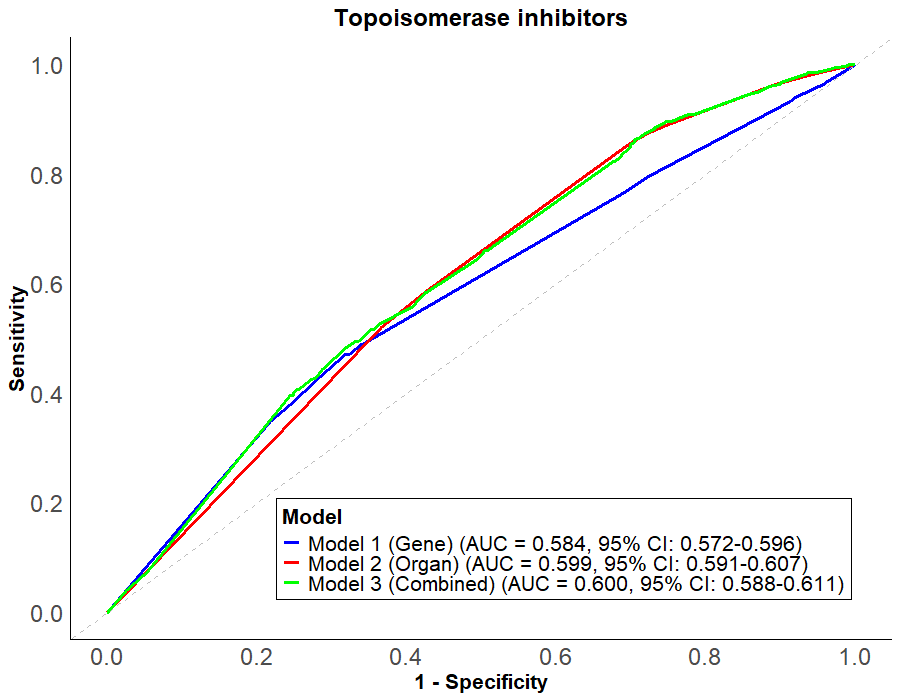
**
